# The *Toxoplasma* protein phosphatase 6 catalytic subunit (TgPP6C) is essential for cell cycle progression and virulence

**DOI:** 10.1371/journal.ppat.1011831

**Published:** 2023-12-13

**Authors:** Qin-Li Liang, Lan-Bi Nie, Hany M. Elsheikha, Ting-Ting Li, Li-Xiu Sun, Zhi-Wei Zhang, Meng Wang, Bao-Quan Fu, Xing-Quan Zhu, Jin-Lei Wang

**Affiliations:** 1 State Key Laboratory for Animal Disease Control and Prevention, Key Laboratory of Veterinary Parasitology of Gansu Province, Lanzhou Veterinary Research Institute, Chinese Academy of Agricultural Sciences, Lanzhou, China; 2 Faculty of Medicine and Health Sciences, School of Veterinary Medicine and Science, University of Nottingham, Loughborough, United Kingdom; 3 Institute of Urban Agriculture, Chinese Academy of Agricultural Sciences, Chengdu, China; 4 Laboratory of Parasitic Diseases, College of Veterinary Medicine, Shanxi Agricultural University, Taigu, China; UTSW: The University of Texas Southwestern Medical Center, UNITED STATES

## Abstract

Protein phosphatases are post-translational regulators of *Toxoplasma gondii* proliferation, tachyzoite-bradyzoite differentiation and pathogenesis. Here, we identify the putative protein phosphatase 6 (TgPP6) subunits of *T*. *gondii* and elucidate their role in the parasite lytic cycle. The putative catalytic subunit TgPP6C and regulatory subunit TgPP6R likely form a complex whereas the predicted structural subunit TgPP6S, with low homology to the human PP6 structural subunit, does not coassemble with TgPP6C and TgPP6R. Functional studies showed that TgPP6C and TgPP6R are essential for parasite growth and replication. The ablation of TgPP6C significantly reduced the synchronous division of the parasite’s daughter cells during endodyogeny, resulting in disordered rosettes. Moreover, the six conserved motifs of TgPP6C were required for efficient endodyogeny. Phosphoproteomic analysis revealed that ablation of TgPP6C predominately altered the phosphorylation status of proteins involved in the regulation of the parasite cell cycle. Deletion of TgPP6C significantly attenuated the parasite virulence in mice. Immunization of mice with TgPP6C-deficient type I RH strain induced protective immunity against challenge with a lethal dose of RH or PYS tachyzoites and Pru cysts. Taken together, the results show that TgPP6C contributes to the cell division, replication and pathogenicity in *T*. *gondii*.

## Author summary

*Toxoplasma gondii* is a protozoan parasite characterized by a highly spatially and temporally coordinated replication process. Some protein phosphatases are conserved among apicomplexan protozoa and regulate numerous cellular and biological processes, such as parasite proliferation, tachyzoite-bradyzoite differentiation and virulence. Here, we identify the role of the putative protein phosphatase 6 of *T*. *gondii* (TgPP6), and found two of its subunits, TgPP6C and TgPP6R, to be vital for the parasite replication and virulence. RHΔ*pp6c* and RHΔ*pp6r* exhibited various degrees of asynchronous division and morphological deformation during endodyogeny compared to wild-type parasites. TgPP6C affected the phosphorylation status of proteins involved in the parasite cell division. We also demonstrated the protective efficacy of immunization of mice using RHΔ*pp6c* against acute and chronic infection by wild-type RH, PYS and Pru strains, respectively. These findings reveal novel roles of TgPP6C in the replication and virulence of *T*. *gondii*.

## Introduction

Members of the phylum Apicomplexa are unicellular, obligate, intracellular protozoan parasites, including *Toxoplasma gondii*, the causative agent of toxoplasmosis [1,2]. *T*. *gondii* has a complex life cycle comprising three main developmental stages, tachyzoites, bradyzoites and oocysts, of distinct phenotypic traits [3]. The pathogenicity of *T*. *gondii* infection is correlated with the rapid proliferation of tachyzoites and the exponential increase in the number of parasites, which result in host cell destruction [4]. Given the limitations of anti-*T*. *gondii* treatment, it is essential to enhance our understanding of the pathogenic mechanisms of *T*. *gondii* in order to identify new therapeutic targets for toxoplasmosis.

*T*. *gondii* tachyzoites replicate by endodyogeny, asexual division process in which two daughter parasite buds develop in the cytoplasm of the parental parasite cell [5–7]. The centrosome is essential for the division of *T*. *gondii*, and its migration from the apical to the basal side of the nuclear envelope marks the beginning of endodyogeny [3]. The maturation of the centrosome is critical for the formation of new daughter parasites and coordination of the budding process [4,8]. In addition, when the parental parasite cell divides, the apicoplast becomes associated with the centrosome, and then become elongated. After replication is completed, the centrosome returns to its original location [9,10]. Another structure critical to the *T*. *gondii* cell cycle is the inner membrane complex (IMC), which is one of the earliest structures to appear during tachyzoite division and plays a role on maintaining the parasite morphology [11]. During endodyogeny, IMC proteins with different functions are synthesized and added to the membrane and cytoskeleton of the daughter buds in a “just-in-time” manner to ensure replication progression [12]. IMC suture proteins are also associated with the cytoskeletal network, which is critical for ensuring parasite morphology and replication synchronization [13,14].

Phosphorylation, one of the most common and important post-translational modifications (PTMs), is reversibly regulated by protein kinases and phosphatases [15]. A considerable proportion of *T*. *gondii* proteins involved in various stages of the parasite lytic cycle (e.g., invasion, replication and egress) and in tachyzoite-bradyzoite differentiation, are differentially phosphorylated [16–18]. Phosphoprotein phosphatases (PPPs) play diverse roles in many biological processes [16]. Our recent study demonstrated that the protein phosphatase 2A (PP2A), a member of *T*. *gondii* PPPs, mediates dephosphorylation, thereby affecting starch metabolism and bradyzoite differentiation [17]. Protein phosphatase 6 (PP6), a member of the PPP family, is indispensable for many organisms including yeast, mammals and plants [19]. The PP6 of *Saccharomyces cerevisiae* and humans is required for cell cycle progression from G1 to S phase but has different substrate specificities [20]. Knock-down of the catalytic subunit of PP6 (PPP6C) in human cells increases the frequency of apoptosis, while the loss of the catalytic subunit of PP6 (SIT4) in *S*. *cerevisiae* causes severe growth defects [21,22]. *Arabidopsis* encodes two PP6 catalytic subunits (FyPP1/FyPP3), whose simultaneous disruption causes significant defects in the plant growth and development [23]. Similar to *S*. *cerevisiae* and humans, *T*. *gondii* has a gene encoding the putative PP6 catalytic subunit (TGGT1_301010, TgPP6C), in addition to a putative regulatory subunit (TGGT1_319930, TgPP6R), and a putative structural subunit (TGGT1_216680, TgPP6S) [16,19]. Although the relative fitness scores according to *T*. *gondii* genome-wide CRISPR screens suggest that TgPP6 may play an essential role in *T*. *gondii* [24], its involvement in the pathogenicity of *T*. *gondii* has not been studied.

In this study, we characterized the putative TgPP6 complex and investigated its role in *T*. *gondii* replication and pathogenicity. Our results show that the catalytic TgPP6C and regulatory TgPP6R subunits, but not the structural subunit TgPP6S, form a complex. Intriguingly, both TgPP6C and TgPP6R interact with the hypothetical protein TGGT1_203150. Functional studies showed that TgPP6C and TgPP6R play an important role in the parasite’s replication by influencing endodyogeny. Comparative phosphoproteomics revealed that the phosphorylation status of many cell cycle associated proteins, especially of IMC components, were significantly altered when TgPP6C was deleted. Mice infection assays showed that deletion of TgPP6C and TgPP6R significantly attenuated *T*. *gondii* virulence, and that immunization of mice with RHΔ*pp6c* induced a mixed Th1/Th2 immune response and conferred protection against acute and chronic infection by the respective wild-type (WT) strains. Overall, our data reveal that TgPP6C plays an essential role in the replication and virulence of *T*. *gondii*.

## Results

### TgPP6 is expressed during the asexual development of *T*. *gondii* tachyzoites

**Fig 1:**
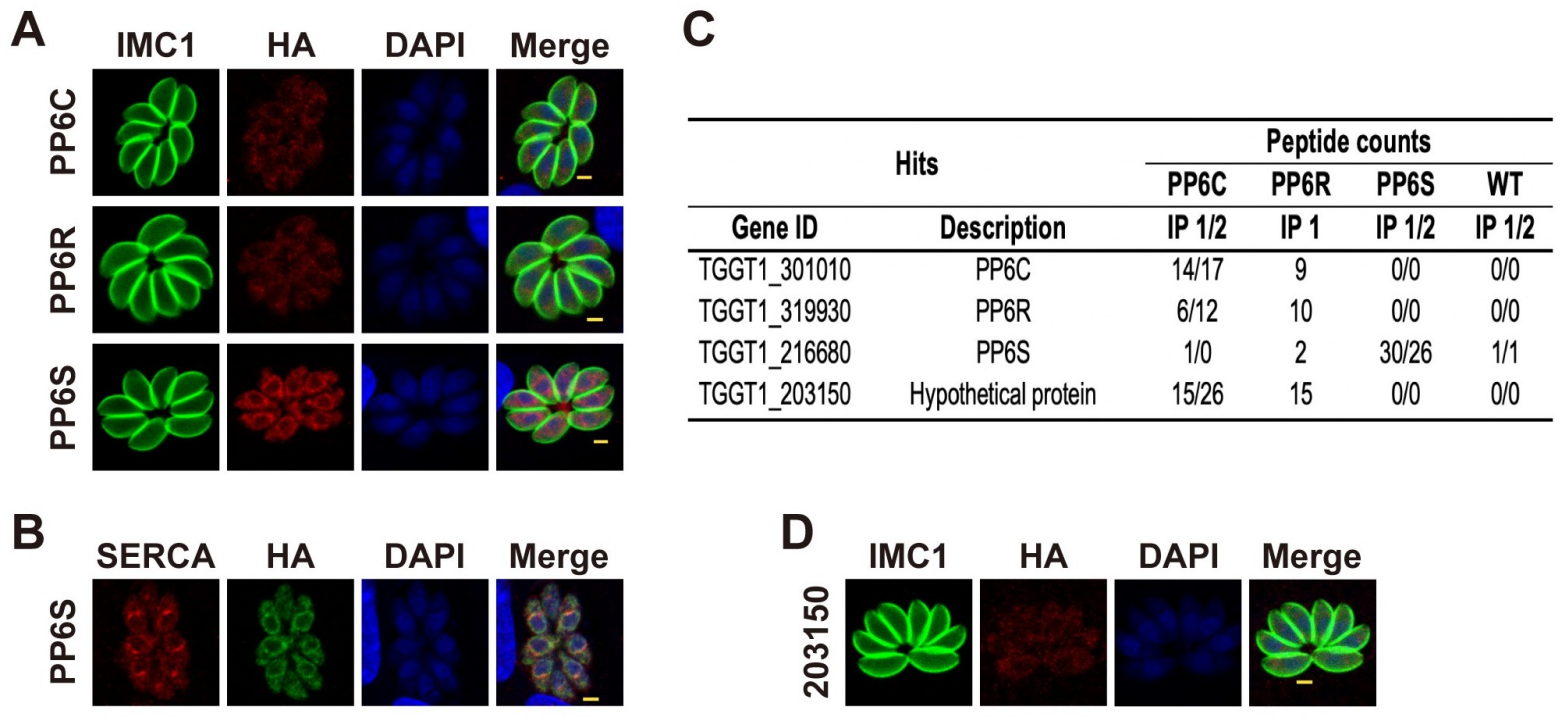
Characterization of the putative PP6 of *Toxoplasma gondii*. (**A**) The subcellular localization of TgPP6C and TgPP6R by endogenous tagging shows two subunits localized in the cytoplasm, while TgPP6S was located in the endoplasmic reticulum. Green, TgIMC1 stain denotes the outline of the parasite. Red, mouse anti-HA antibody. Scale bars: 2 μm. (**B**) IFA shows TgPP6S::6HA (green) colocalized with the endoplasmic reticulum protein TgSERCA (red). Scale bars: 2 μm. (**C**) Immunoprecipitation (IP) assays were performed to identify TgPP6::6HA subunits (TgPP6C, TgPP6R and TgPP6S) interacting proteins, WT strain as a control. Selected hits identified by LC-MS analyses are shown and the numerical annotations of each gene show the count of unique peptides originating from the MS analysis that matched the corresponding hits. A full list of the hits is provided in S1 Table. (**D**) TGGT1_203150::6HA parasites were stained with antibodies against TgIMC1 (green) and HA (red). TGGT1_203150 was located in the cytoplasm of the parasite. Scale bars: 2 μm.

Based on the ToxoDB database (https://toxodb.org), *T*. *gondii* PP6 has a potential catalytic subunit (TgPP6C, TGGT1_301010), a regulatory subunit (TgPP6R, TGGT1_319930) and a structural subunit (TgPP6S, TGGT1_216680) [16]. To determine the localization of the putative subunits of TgPP6, we generated strains (TgPP6C::6HA, TgPP6R::6HA and TgPP6S::6HA) with a C-terminal epitope tag in *T*. *gondii* type I RHΔ*ku80* (WT) strain by using CRISPR-Cas9 (S1 Fig). We found that TgPP6C and TgPP6R were localized in the cytoplasm of tachyzoites, whereas TgPP6S was observed in the endoplasmic reticulum (ER) (Fig 1A). To further confirm the localization of this protein, we co-localized TgPP6S with the ER marker sarcoplasmic-endoplasmic reticulum calcium ATPase (TgSERCA) [25], and found that TgPP6S is indeed located in the ER of tachyzoites (Fig 1B).

To investigate potential interactions between the putative TgPP6 protein subunits in *T*. *gondii*, we immunoprecipitated lysates generated from the TgPP6C::6HA, TgPP6R::6HA and TgPP6S::6HA strains with anti-HA-conjugated magnetic beads, which were subjected to LC-MS analysis. The immunoprecipitation (IP) results were consistent with TgPP6C and TgPP6R interacting with each other, while TgPP6S did not appear to interact with TgPP6C or TgPP6R because no TgPP6C or TgPP6R peptides were detected in the TgPP6S::6HA group. This result suggests that TgPP6S may not be part of the TgPP6C and TgPP6R complex (Fig 1C and S1 Table). The low homology between TgPP6S and human PP6 structural subunits further implies that TgPP6S may function in a distinct way from the other TgPP6 subunits. Interestingly, both TgPP6C::6HA and TgPP6R::6HA groups identified the hypothetical protein TGGT1_203150 as a potential interaction partner, suggesting that TGGT1_203150 may form a heterotrimer with TgPP6C and TgPP6R (Fig 1C). Subsequently, a TGGT1_203150::6HA strain was generated, and immunofluorescence staining confirmed its subcellular localization in the parasite cytoplasm (Fig 1D).

### TgPP6C and TgPP6R are indispensable for *T*. *gondii* replication

**Fig 2:**
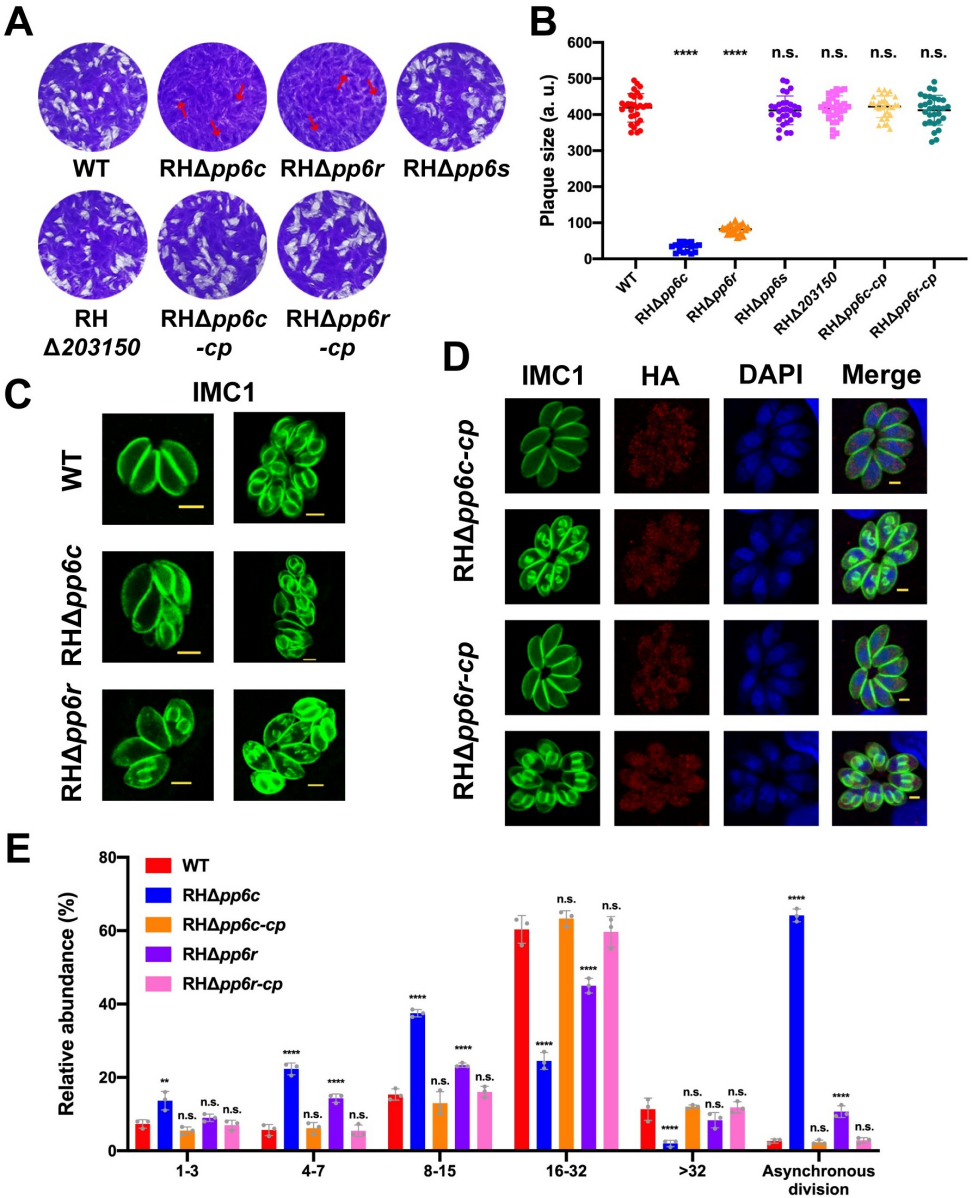
Phenotypic analyses of TgPP6 subunits. (**A**) Representative images of the plaque assays of the indicated strains grown for 7 days, showing that the ability of RHΔ*pp6c* and RHΔ*pp6r* to form plaques is almost completely inhibited. The arrows indicate the plaques formed by RHΔ*pp6c* and RHΔ*pp6r*. (**B**) The relative size of the plaques produced by each strain. Data represents mean ± SD of three independent experiments and statistical differences were analyzed by the unpaired *t*-test. ****P < 0.0001; n.s., not significant. (**C**) Deletion of TgPP6C and TgPP6R resulted in tachyzoites with dysregulated endodyogeny and morphological defects. Green, TgIMC1. Scale bar: 2 μm. (**D**) The validation of the complemented strain RHΔ*pp6c-cp* and RHΔ*pp6r-cp* shows that both strains regained their normal endodyogeny. Green, TgIMC1. Red, HA. Scale bar: 2 μm. (**E**) Intracellular replication of the WT, RHΔ*pp6c*, RHΔ*pp6c-cp*, RHΔ*pp6r* and RHΔ*pp6r-cp* strains. The intracellular replication rate and the percentage of asynchronous division of RHΔ*pp6c* and RHΔ*pp6r* were significantly different from that of the WT strain. Data are mean ± SD from three independent experiments, > 200 vacuoles were counted per replicate and statistical differences were analyzed by a two-way ANOVA. **P = 0.0012; ****P < 0.0001; n.s., not significant.

To elucidate the biological significance of each putative subunit of TgPP6 to parasite fitness, CRISPR-Cas9-mediated gene homologous recombination was used to disrupt TgPP6C, TgPP6R, TgPP6S and TGGT1_203150, respectively (S2A Fig). Single knock-out clones were screened using diagnostic PCRs, with PCR3 and PCR5 confirming the replacement of homologous fragments. These PCRs amplified a ~1200–1600 bp fragment, which was absent in the WT strain (S2B Fig). In contrast, the expected coding sequence of PCR4 (~ 600–800 bp) was amplified in the WT strain but was absent in the RHΔ*pp6* strains (S2B Fig). To explore the roles of these four genes in the growth of parasites *in vitro*, plaque assays were performed using WT and knock-out strains. There was no significant difference in the size and number of plaques formed by the WT, RHΔ*pp6s* and RHΔ*203150*, however RHΔ*pp6c* and RHΔ*pp6r* strains formed smaller plaques in HFFs compared to those in WT after 7 days of incubation (Fig 2A and 2B), suggesting that both TgPP6C and TgPP6R are important for the parasite growth. This result is consistent with the low phenotypic score (–4.05 for TgPP6C and –3.76 for TgPP6R) in *T*. *gondii* genome-wide CRISPR-Cas9 screens [24]. To further confirm that the growth defect of the parasite was caused by the deletion of TgPP6C and TgPP6R, RHΔ*pp6c* or RHΔ*pp6r* strains were complemented by inserting TgPP6C or TgPP6R fused with a C-terminal 3×HA epitope tag driven by β-tubulin promoter into the UPRT locus using CRISPR-Cas9 directed gene integration (S2C Fig). Complemention of RHΔ*pp6c* strain with TgPP6C (RHΔ*pp6c-cp*) or RHΔ*pp6r* strain with TgPP6R (RHΔ*pp6r-cp*) successfully restored the parasite growth.

The significant reduction in plaque formation suggests some defects in one or multiple stages in the parasite lytic cycle. Therefore, we examined the invasion, intracellular replication and egress properties of RHΔ*pp6c* and RHΔ*pp6r*. The deletion of TgPP6C and TgPP6R did not significantly affect the egress of tachyzoites, but slightly affected their ability to invade host cells (S2D and S2E Fig). Interestingly, both RHΔ*pp6c* and RHΔ*pp6r* exhibited a significant reduction in replication efficiency compared with WT strain. In addition, an abnormal phenotype with asynchronous division of the intracellular tachyzoites was observed in the RHΔ*pp6c* strain (Fig 2C). While a small number of the RHΔ*pp6r* strain also exhibited this asynchronous division, it was less pronounced than that seen in the RHΔ*pp6c* strain (Fig 2C). Based on this results, the study was we focused on TgPP6C. When the number of tachyzoites in the parasitophorous vacuoles (PVs) reached “2”, the division of certain RHΔ*pp6c* tachyzoites became asynchronous and when it divided further to “4”, or more (i.e., 4–8, 8–16, 16–32 and > 32 divisions) the tachyzoites were unable to form normal "rosettes". This abnormal phenotype was restored to the wild-type level in the complemented strain (Fig 2D).

Therefore, we infer that RHΔ*pp6c* and RHΔ*pp6r* mainly affects the tachyzoite replication. To verify our hypothesis, intracellular replication assays were performed. We cultured the parasites for a longer period of time compared to normal replication assays because most of the PVs of RHΔ*pp6c* and RHΔ*pp6r* strains contained only two or four tachyzoites 24 h post infection. We quantified the replication rate of RHΔ*pp6c* and RHΔ*pp6r*, and quantified the asynchronous division events which appeared in PVs containing > 2 tachyzoites. Thus, all the strains were cultured for 32 h, the proportion of PVs with 1–3 (13.67 ± 2.52%), 4–7 (22.33 ± 1.61%) and 8–15 (37.5 ± 1%) tachyzoites was significantly higher in the RHΔ*pp6c* strain compared to the WT strain and RHΔ*pp6c-cp* strain, while the proportion of PVs containing 16–32 (24.5 ± 2.29%) and > 32 (2 ± 0.87%) tachyzoites were significantly lower in RHΔ*pp6c* strain compared to the WT and RHΔ*pp6c-cp* strains (Fig 2E). Additionally, the proportion of irregular PVs (i.e., containing asynchronously dividing parasites) of RHΔ*pp6c* (64.17 ± 1.76%) was significantly higher compared to the other strains (Fig 2E). As for the RHΔ*pp6r* strain, the replication ability was also significantly reduced, and the anomalous division (10.67 ± 1.61%) occurred on a notably smaller scale compared to that of the RHΔ*pp6c* strain (Fig 2E).

Given the crucial role of TgPP6C and TgPP6R in parasite repliaction, their functions were also examined using a conditional protein depletion approach. To achieve this, the endogenous TgPP6C and TgPP6R genes were tagged with an indole-3-acetic acid (IAA)-inducible degradation domain (mAID-6HA) at the C-terminus in the RHΔ*ku80*::TIR1 (RH::TIR1) strain. As expected, after treatment with IAA, PP6C-mAID and PP6R-mAID parasites exhibited growth defects, asynchronous division and reduced replication rate, similar to that observed in RHΔ*pp6c* and RHΔ*pp6r* (S3 Fig). These results show the significant involvement of TgPP6C and TgPP6R in parasite replication.

### TgPP6C is required for synchronous daughter budding and mitosis

**Fig 3:**
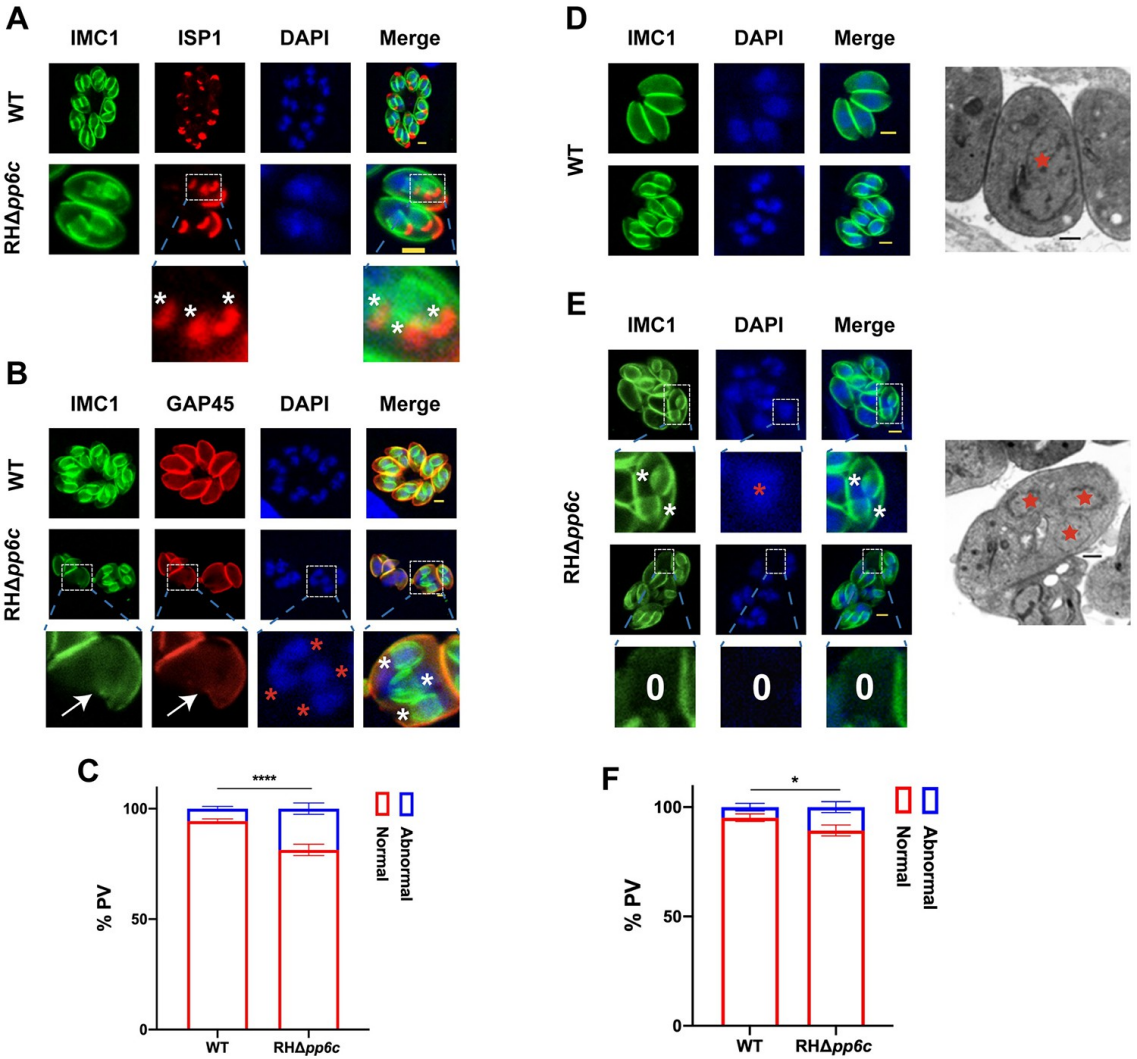
TgPP6C plays a critical role in daughter budding and nuclear segregation. (**A**) to (**C**) Immunofluorescence staining shows that RHΔ*pp6c* exhibits significant increase in endopolygeny. Green, TgIMC1. Red, TgISP1/GAP45. White asterisks denote daughter buds. Red asterisk indicates the parasite nucleus. Arrows indicate the deformed parasite. Scale bar: 2 μm. Data are mean ± SD from three independent experiments (> 200 vacuoles were counted per replicate), and statistical differences were analyzed by a two-way ANOVA; ****P < 0.0001. (**D**) to (**F**) Immunofluorescence staining and electron micrographs show that lack of TgPP6C resulted in marked defects in the segregation of the parasite nucleus. White asterisks, red asterisks and white numbers denote daughter buds, nuclei and number of nuclei, respectively. TgIMC1 and DAPI stains were used to stain the outline and nuclei (DNA) of the parasites, respectively. Scale bar: IFA, 2 μm; Electron microscopy, 1 μm. Data shown in the graphs represent the mean ± SD of three independent experiments (> 200 vacuoles were counted per replicate) and statistical differences were analyzed by a two-way ANOVA; *P = 0.0204.

As explained earlier, TgPP6C plays an indispensable role in promoting the growth of type I RH parasites, particularly during their replication stage, while concurrently affecting endodyogeny. To assess endodyogeny changes in TgPP6C knock-out parasites, we stained intracellular parasites separately with IMC1/ISP1 (IMC sub-compartment protein 1) and IMC1/GAP45 combinations. TgISP1, as an early progenitor IMC protein, is present at the apical cap of both parental and progeny parasites, whereas the expression of TgGAP45 is confined solely to the parental parasite. We noted an increase in the proportion (18.67 ± 2.57%) of RHΔ*pp6c* parasites exhibiting endopolygeny, together with impairment in the divisional capacity of some parental parasites (Fig 3A to 3C). However, absence of PP6C did not affect the integrity of the IMCs and pellicles. Additionally, in up to 10.67 ± 2.47% of PVs of RHΔ*pp6c* strain (Fig 3B, 3D to 3F), nuclear segregation defects (e.g. abnormal segregation or inability to segregate) were observed. These nuclear phenotypic chnages were also observed in RHΔ*pp6c* by electron microscopy (Fig 3E). The detection of multinucleated parasites confirmed that some RHΔ*pp6c* undergo endopolygeny replication.

**Fig 4:**
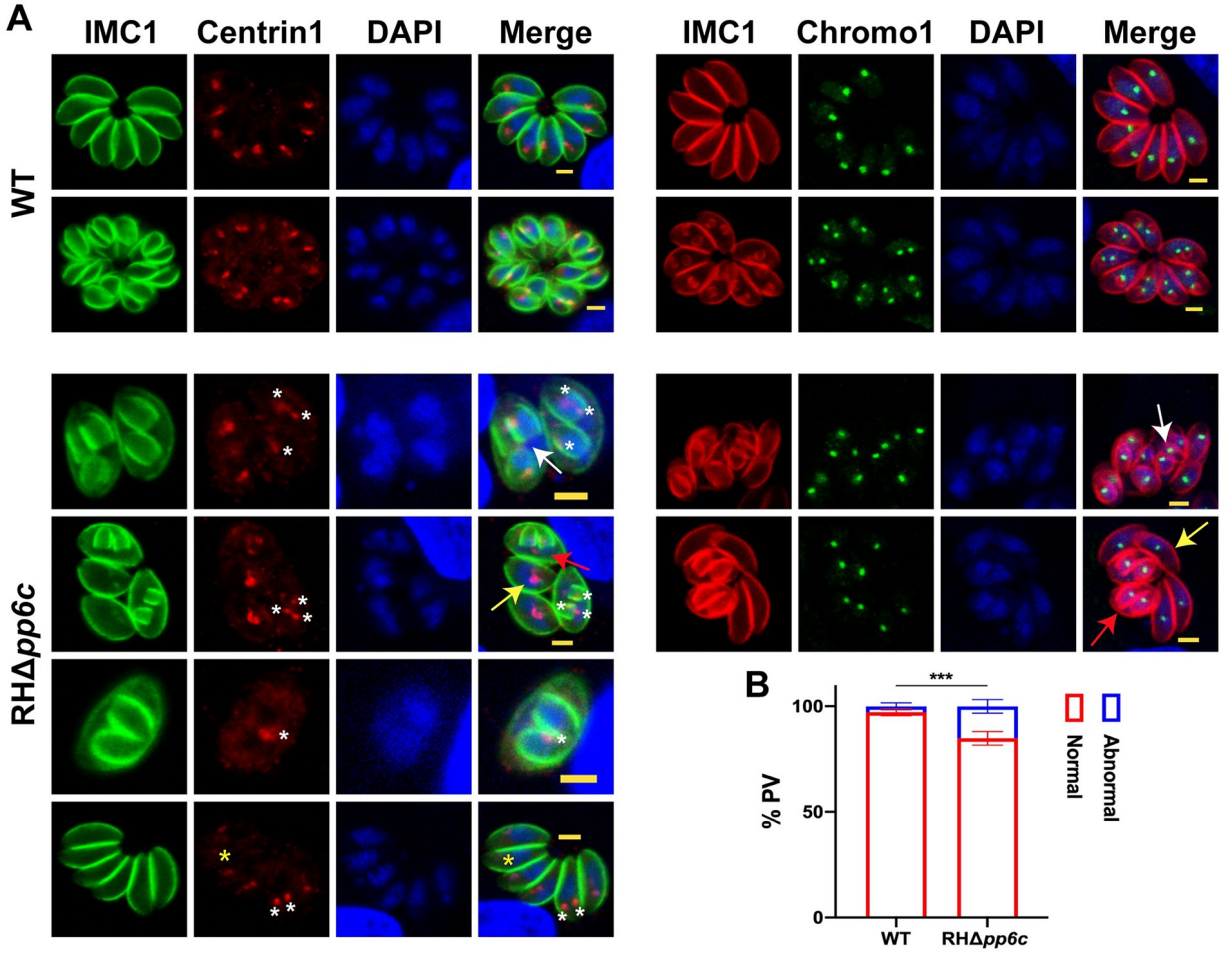
TgPP6C is important for the stability of centrosome. (**A**) Immunofluorescence staining shows that RHΔ*pp6c* harboring the duplicated, awaiting to be duplicated or over-duplicated centrosome and centromere within an abnormal PV as revealed by Centrin1 and Chromo1 markers, respectively. White and yellow asterisks indicate a centrosome and a missing centrosome, respectively. Red, yellow and white arrows denote the duplicated, awaiting to be duplicated and over-duplicated centrosome and centromere, respectively. Red, TgIMC1/TgCentrin1. Green, TgIMC1/TgChromo1. Scale bar: 2 μm. (**B**) Data are mean ± SD from three independent experiments (> 200 vacuoles were counted per replicate), and statistical differences were analyzed by a two-way ANOVA; ***P = 0.0007.

*T*. *gondii*’s cell cycle has a precisely timed and strictly regulated sequence of subcellular divisions [10]. The initial organelle to undergo duplication is the centrosome, which plays a key role in the division of *T*. *gondii* [4]. We used TgCentrin1 as a centrosome marker protein to examine its potential alterations in the absence of TgPP6C [4]. By staining TgCentrin1, we discovered that most TgCentrin1 exhibited a persistent inclination towards division, harboring the duplicated, awaiting to be duplicated or over-duplicated entities within abnormal PVs (Fig 4A). Further examination of tachyzoites revealed discrete, brightly marked centrosomes in close proximity to the nucleus in the WT strain, whereas the centrosomes in RHΔ*pp6c* tachyzoites (comprising 15.17 ± 3.22%) exhibited anomalies (Fig 4A and 4B). These irregularities include impaired duplication within the parental parasites or instances where the daughter parasite nuclei were associated with duplicated centrosomes (Fig 4A and 4B). Considering the role of TgChromo1, which remains near the centrosome and centrocone throughout the cell cycle, it functions as a centromere marker [26]. In agreement with our reasoning, staining of TgChromo1 closely mirrored earlier findings from TgCentrin1 staining, however, the stability of centromere was not significantly altered in RHΔ*pp6c* (Fig 4A). These results indicate that TgPP6C may contribute to the stability of the centrosome.

Next, we examined the effect of TgPP6C deletion on other organelles, including rhoptries, dense granules, apicoplast, mitochondria and micronemes. Interestingly, tachyzoites continued to replicate despite considerable morphological defects (e.g., enlarged paternal parasites or abnormal endodyogeny) caused by TgPP6C deletion (S4 Fig). However there were no obvious effects on the other *T*. *gondii* organelles. These results suggest that TgPP6C plays a role in the modulation of *T*. *gondii*’s cell cycle-specific expression pattern, and its deletion affects parasite replication, from the inception of the S phase onwards.

### TgPP6C core motifs are required for endodyogeny and function

**Fig 5:**
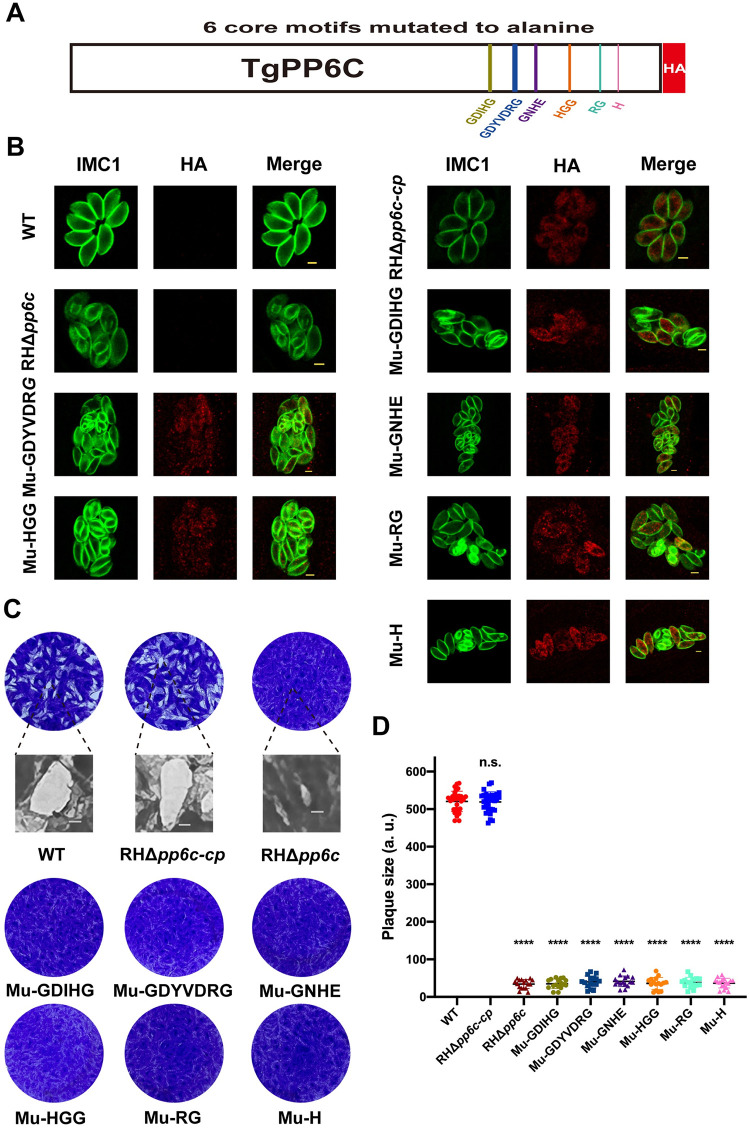
The TgPP6C core motif is essential for its function. (**A**) Gene model for TgPP6C with the six predicted core catalytic motifs mutated to alanine. (**B**) Immunofluorescence staining revealed that six core motifs of TgPP6C are essential for *T*. *gondii* endodyogeny. Green, TgIMC1. Red, HA. Scale bar: 2 μm. (**C**) to (**D**) The size and number of plaques formed by RHΔ*pp6c* and mutant strains with each core motif mutated to alanine were significantly reduced compared with the WT and RHΔ*pp6c-cp* strains. Data represents mean ± SD of three independent experiments and statistical differences were analyzed by the unpaired *t*-test. The indicated strains were compared with WT. ****P < 0.0001; n.s., not significant. Scale bar: 0.5 mm.

TgPP6C possesses core motifs GDIHG, GDYVDRG, GNHE, HGG, RG and H, which are conserved in eukaryotes [15,16]. To assess the role of the predicted TgPP6C core motifs, we individually introduced mutations at every amino acid position within the six core motifs, by substituting them with alanine (MU-GDIHG/AAAAA, MU-GDYVDRG/AAAAAAA, MU-GNHE/AAAA, MU-HGG/AAA, MU-RG/AA and MU-H/A) in the vector of the complemented TgPP6C, which includes a C-terminus 3×HA epitope tag and is driven by β-tubulin promoter (Figs 5A and S2B). Each target fragment was inserted into the UPRT locus of RHΔ*pp6c* by CRISPR-Cas9-mediated gene integration, and the clonal strains were successfully obtained by chloramphenicol selection and limited dilution. The mutation of each of the six motifs failed to complement the parasite’s phenotype, and the division remained asynchronous, indicating that all the TgPP6C motifs are indispensable for the intracellular division of *T*. *gondii*, but they appear to play no or minor role in TgPP6C localization (Fig 5B). Interestingly, RHΔ*pp6c* and each mutant produced hardly visible plaques, which were significantly smaller than those of the WT and RHΔ*pp6c-cp* (Fig 5C and 5D). These results show that mutation in any of the six motifs of TgPP6C could not rescue the TgPP6C knock-out, suggesting critical role of these motifs in the parasite’s endodyogeny.

### Disruption of TgPP6C alters phosphorylation status of many proteins related to parasite cell cycle and division

**Fig 6:**
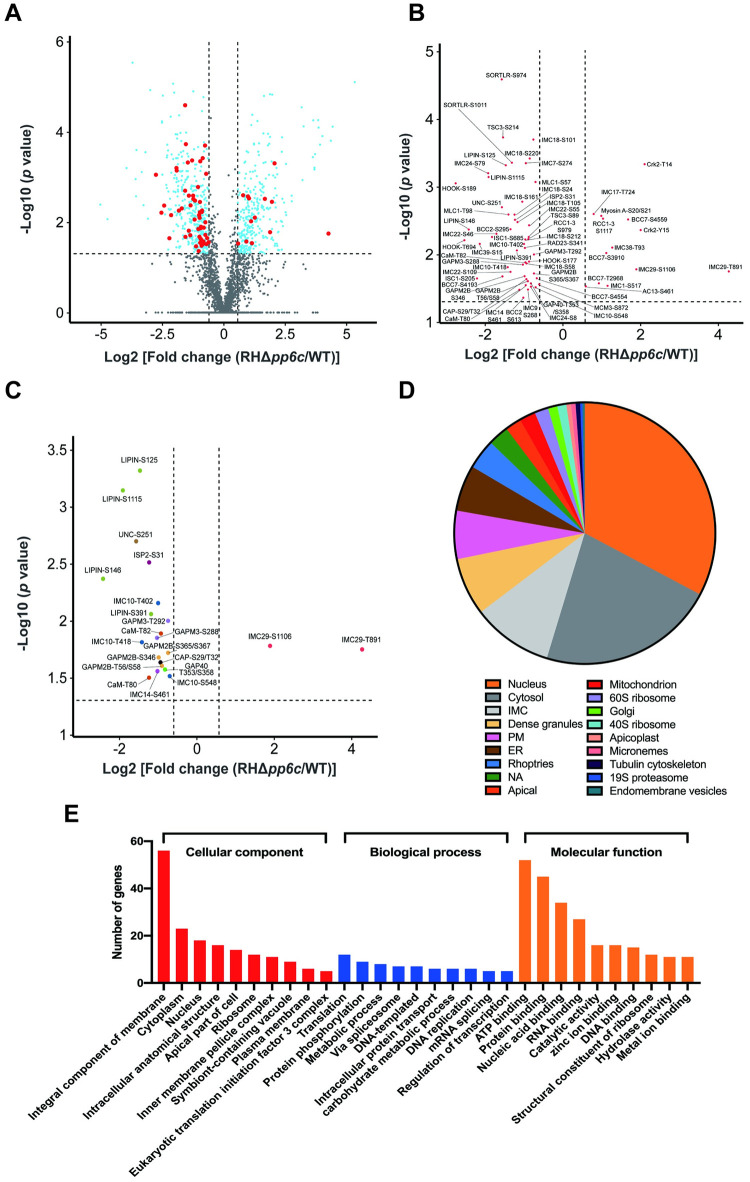
Functional annotation of the significantly regulated phosphoproteins. (**A**) to (**C**) Volcano plots showing the significant fold changes of the phosphopeptides from RHΔ*pp6c* strain compared with WT strain. Red and blue dots represent up-regulated (fold changes > 1.5 with *P* < 0.05) or down-regulated (fold changes < –1.5 with *P* < 0.05), and gray dots represent non-differentially regulated proteins. Proteins with predicted or known roles in chromosome organization, cell cycle progression and cell division are shown as red dots. Those proteins are plotted in (**B**), and some of them have more than one differentially phosphorylated site. Eleven phosphoproteins that have been previously shown to be associated with cell division during the tachyzoite lytic cycle were plotted in (**C**) and each phosphoprotein was shown in a different color. (**D**) The percentage of differential phosphoproteins relative to the total number of differential phosphoproteins, according to their predicted localization. (**E**) The top 10 significantly enriched GO terms of the differentially regulated phosphoproteins in the cellular component, biological process, and molecular function categories of RHΔ*pp6c* vs WT.

To identify phosphorylation events mediated by TgPP6C, quantitative phosphoproteomic analysis was performed on TgPP6C knock-out parasites compared with the WT strain using label-free phosphoproteomics. This analysis identified 434 differentially regulated proteins between RHΔ*pp6c* and WT, of those 226 proteins were upregulated in RHΔ*pp6c* and 208 were upregulated in WT, as defined by a *P* < 0.05 and fold-change of < –1.5 or > 1.5 (S2 Table). Additionally, 8,748 phosphosites were identified in 2,390 proteins (S3 Table). To determine the changes in the phosphorylated proteins between RHΔ*pp6c* and WT, we normalized the level of phosphorylated proteins relative to the level of all the identified phosphorylated peptides (2581 phosphorylation sites and 706 proteins) (S3 Table). After normalization, 777 unique phosphorylation sites on 375 *T*. *gondii* proteins exhibited significantly altered abundance in RHΔ*pp6c*. Of these, 350 phosphopeptides on 162 phosphoproteins were upregulated, and 427 phosphopeptides on 213 phosphoproteins were downregulated in RHΔ*pp6c* (Fig 6A and S3 Table). The down-regulated phosphopeptides may be a secondary effect to the loss of TgPP6C, which may have led to activation of other phosphatases or suppressing kinases.

A large number of proteins involved in the regulation of the cell cycle and most of the IMC proteins were differentially regulated in RHΔ*pp6c* strain compared with WT strain (Fig 6A to 6C). In addition, deletion of TgPP6C resulted in both upregulated and downregulated phosphorylation sites in four proteins (TGGT1_265250, TGGT1_258870A, TGGT1_269180 and TGGT1_220240). Both TGGT1_265250 and TGGT1_269180 have predicted RNA binding activities, while TGGT1_258870A and TGGT1_220240 (GRA31) are integral components of the membrane.

An in-depth look at differentially phosphorylated proteins between RHΔ*pp6c* strain and WT strain revealed that many of them are predicted or known to play roles in chromosome organization, cell cycle progression and cell division (Fig 6B). Specifically, IMC-related proteins accounted for the majority of the differentially phosphorylated proteins, including (TGGT1_235340, ISC1), (TGGT1_237820, ISP2), (TGGT1_220900, AC13), (TGGT1_231070, BCC2), (TGGT1_311230, BCC7), (TGGT1_230850, TSC3), (TGGT1_249850, GAP40), (TGGT1_206690, GAPM2B), (TGGT1_271970, GAPM3), (TGGT1_235470, Myosin A), (TGGT1_257680, MLC1), (TGGT1_231640, IMC1), (TGGT1_222220, IMC7), (TGGT1_226220, IMC9), (TGGT1_230210, IMC10), (TGGT1_260540, IMC14), (TGGT1_286580, IMC17), (TGGT1_295360, IMC18), (TGGT1_316340, IMC22) (TGGT1_258470, IMC24), (TGGT1_243200, IMC29), (TGGT1_293360, IMC38), and (TGGT1_255420, IMC39) (Fig 6B).

Focusing on the function of selected differential phosphoproteins, Cdk-related kinase 2 (TGGT1_218220, Crk2) plays a role in the regulation of the tachyzoite G1 phase [27]. Its absence induces the arrest of the G1 phase. Moreover, upon deletion of TgPP6C, two proteins were differentially phosphorylated (TGGT1_289100, HOOK and TGGT1_290160, SORTLR), which are involved in the parasite invasion, egress and virulence [28,29]. Some differentially phosphorylated proteins contribute to the division of *T*. *gondii*, including IMC proteins (IMC10, IMC14 and IMC29), IMC related proteins (ISP2, GAP40, GAPM2B and GAPM3), phosphatidic acid phosphatase (TGGT1_230690, LIPIN), myosin-specific co-chaperone of the UCS family (TGGT1_249480, UNC), cyclase associated protein (TGGT1_310030, CAP) and calmodulin (TGGT1_249240, CaM) [30–42] (Fig 6C and Table 1). The nucleus, cytosol and IMC were the main predicted locations for most of the differentially phosphorylated proteins based on the spatial data obtained from hyper LOPIT [43] (Fig 6D and S4 Table).

**Table 1:** List of the differentially phosphorylated proteins involved in tachyzoite division as revealed by phosphoproteome analysis.

Gene ID	Annotation	GWCS^a^	Localization
TGGT1_249240	CaM	-5.82	Apical, basal and cytoplasm [30]
TGGT1_243200	IMC29	-3.95	IMC [31,32]
TGGT1_230690	LIPIN	-3.74	Cytoplasm and ER [33]
TGGT1_249480	UNC	-3.39	Cytoplasm [34]
TGGT1_310030	CAP	1.17	Apical and cytoplasm [35,36]
TGGT1_230210	IMC10	-4.7	IMC [37,38]
TGGT1_237820	ISP2	-2.04	IMC [39]
TGGT1_249850	GAP40	-3.46	IMC [40,41]
TGGT1_206690	GAPM2B	0.44	IMC [41]
TGGT1_271970	GAPM3	-2.6	IMC [41]
TGGT1_260540	IMC14	1.75	IMC [42]

^a^Phenotype score in a genome-wide CRISPR/Cas9 screen.

We performed GO enrichment analysis to identify the function of the 375 differentially abundant phosphoproteins (Fig 6E). Prominent among the enriched GO terms, within the cellular components, are those associated with proteins residing in membrane-bound organelles, cytoplasm, nucleus and diverse protein-containing complexes (Fig 6E). In the biological process category, translation, protein phosphorylation and metabolic processes, were enriched (Fig 6E). In the molecular function category, the GO terms binding, catalytic activity and the structural constituents of the ribosome were enriched in the significantly up- or down-regulated proteins (Fig 6E). These results show that multiple cell cycle-associated phosphoproteins are differentially regulated in RHΔ*pp6c*, and likely contribute to abnormal endodyogeny in RHΔ*pp6c* mutant tachyzoites.

### RHΔ*pp6c* elicits protective immunity against acute and chronic toxoplasmosis

**Fig 7:**
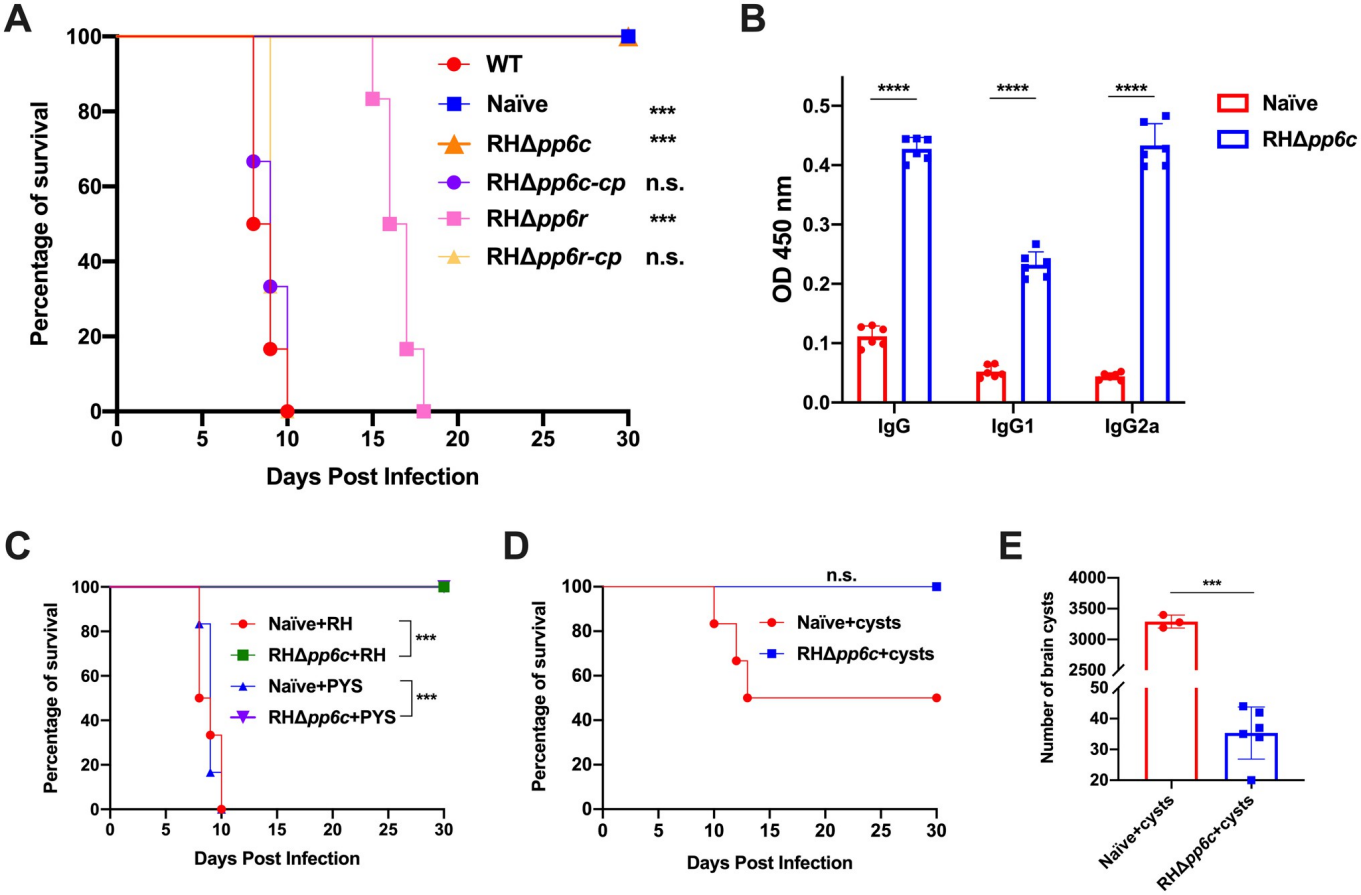
Immunization of RHΔ*pp6c* protects mice against acute and chronic infection by *T*. *gondii*. (**A**) Mice in each group (6 mice/strain) were intraperitoneally (i.p.) injected with 100 tachyzoites of the indicated strains and monitored for 30 days. All RHΔ*pp6c*-infected and control (naïve) mice remained alive without any signs of illness and RHΔ*pp6r*-infected mice had significantly prolonged survival. However, all mice infected with WT, RHΔ*pp6c-cp* and RHΔ*pp6r-cp* reached their humane endpoint within 8–10 days. Statistical significance were tested by log rank Mantel-Cox test. ***P = 0.0005, n.s., not significant. (**B**) The levels of total IgG and IgG isotypes (IgG1 and IgG2a) were determined in the serum of mice immunized with 200 RHΔ*pp6c* tachyzoites (6 mice/strain). Analysis was performed using ELISA at 30 days post-immunization, and the results show that RHΔ*pp6c*-immunized mice developed a high level of IgG and IgG isotypes compared with the naïve mice. Data represnet mean ± SD and statistical differences were analyzed by a two-way ANOVA. ****P < 0.0001. (**C**) 30 days after immunization with RHΔ*pp6c*, mice were injected i.p. with 1000 tachyzoites of RH or PYS (6 mice/strain) and monitored for 30 days. Survival curves indicated that RHΔ*pp6c*-immunized mice were protected against challenge with RH or PYS compared with the naïve mice. Mice in the naïve group reached their humane endpoint within 8–10 days. Statistical significance was examined by log rank Mantel-Cox test. ***P = 0.0007 (Naïve+RH vs. RHΔ*pp6c*+RH), ***P = 0.0006 (Naïve+PYS vs. RHΔ*pp6c*+PYS). (**D**) 30 days after immunization with RHΔ*pp6c*, mice (6 mice/strain) were orally gavaged with 20 Pru cysts and monitored for 30 days. No morbidity and death were detected in RHΔ*pp6c*-immunized mice. However, half of the naïve mice have succumbed to illness and reached their humane endpoint between 10–13 days post challenge. The level of significance was analyzed by log rank Mantel-Cox test. n.s., not significant. (**E**) Parasite cyst burden was quantified in the brain of mice that remained alive at 30 days post infection (*n* = 3 for naïve and *n* = 6 for RHΔ*pp6c*). The cysts in the brain of the RHΔ*pp6c*-immunized mice were significantly lower than that in the naïve mice. Data represnet mean ± SD and Statistical differences were analyzed by the unpaired *t*-test. ***P = 0.0003.

To determine the impact of TgPP6 deficiency on type I RH virulence, groups (*n* = 6/group) of Kunming mice were intraperitoneally (i.p.) injected with 100 tachyzoites in 200 μL phosphate-buffered saline (PBS) of the indicated strains (Fig 7A). Mice in the control (naïve) group were only injected with 200 μL PBS. In congruence with the *in vitro* parasite proliferation results, all mice infected by WT, RHΔ*pp6c-cp* and RHΔ*pp6r-cp* parasites reached their humane end-point within 8–10 days (Fig 7A). In contrast, a substantial extension in the survival was observed in mice infected by RHΔ*pp6r* (Fig 7A). Notably, mice infected by RHΔ*pp6c* remained healthy and did not exhibit any clinical signs (Fig 7A). Anti-*T*. *gondii* IgG and IgG subclasses (IgG1 and IgG2a) were analyzed at day 30 post-immunization with 200 RHΔ*pp6c* tachyzoites to evaluate the Th1/Th2 polarization. The levels of anti-*T*. *gondii* IgG, IgG1 and IgG2a increased significantly in the immunized mice compared with mice in the naïve group (Fig 7B). These results suggest that RHΔ*pp6c* induced a mixed anti-*T*. *gondii* Th1/Th2 response. In addition, IgG2a level was higher relative to IgG1, suggesting that RHΔ*pp6c* induced a Th1-biased antibody response.

To explore whether RHΔ*pp6c* confers protection against acute and chronic infection by *T*. *gondii*, mice were i.p. injected with a single dose of 200 RHΔ*pp6c* tachyzoites in 200 μL PBS or mock-immunized with 200 μL of PBS as the naïve group. Thirty days after immunization, six immunized mice per group were challenged with 10^3^ tachyzoites of type I strain RH or ToxoDB#9 strain PYS, and mouse survival was recorded for 30 days. The results showed that mice in the naïve group reached their humane endpoint within 8–10 days, whereas mice immunized with RHΔ*pp6c* did not exhibit signs of illness for 30 days after challenge by the WT strains (Fig 7C). We also tested the protective efficacy of immunization with RHΔ*pp6c* against chronic infection by orally challenging immunized mice with cysts (20 cysts/mice) of type II Pru strain 30 days post-immunization. As anticipated, 50% of mice in the naïve group succumbed to illness and reached their humane endpoint between 10–13 days post infection, however mice immunized with 200 RHΔ*pp6c* tachyzoites remained alive and did not develop illness (Fig 7D). Parasite cyst burden in the survied mice was counted after 30 days of infection. The number of cysts in the naïve+challenged mice (3,290 ± 105) was significantly higher than that in the RHΔ*pp6c*-immunized+challenged mice (35 ± 8) (Fig 7E). Taken together, these results showed that immunization with low doses of RHΔ*pp6c* conferred significant protection against type I (RH) and ToxoDB#9 (PYS) strains and type II Pru cysts challenge and elicited a mixed anti-*T*. *gondii* Th1/Th2 responses, with a shift toward Th1-mediated cellular immunity axis.

## Discussion

Central to the virulence of *T*. *gondii* infection is the secretion of many effector proteins that mediate host cell invasion, replication, nutrient acquisition and immune evasion. Here, we sought to expand the repertoire of identified virulence factors and investigate the role of PP6 in the *T*. *gondii* lytic cycle. PP6 of the PPP family of protein serine/threonine phosphatases (PSPs) is an evolutionarily conserved and ubiquitously expressed in eukaryotes, and is most closely related to protein phosphatase 2A (PP2A) and protein phosphatase 4 (PP4) [15,44]. In analogy to TgPP2A structure, TgPP6 is predicted to consist of catalytic (TgPP6C), regulatory (TgPP6R) and structural (TgPP6S) subunits [16]. Immunoprecipitation analysis suggested that TgPP6C and TgPP6R may form heterotrimers with the hypothetical protein TGGT1_203150, but not with the predicted TgPP6S. According to the structural analysis in ToxoDB, TGGT1_203150 exhibits homology to the DNA-directed RNA polymerase II subunit rpb1, but lacks any homology to the structural subunit of human PP6. The potential assembly of a complex involving TgPP6C, TgPP6R and TGGT1_203150, warrants further investigation. Although TgPP6S and TGGT1_203150 were dispensable for *T*. *gondii* growth, respective deletion of TgPP6C and TgPP6R resulted in marked growth defects and anomalous endodyogeny.

In the intermediate host, *T*. *gondii* tachyzoites divide by endodyogeny through an intricate internal budding process that results in the formation of two daughter parasites per division [42]. Budding is a hallmark of apicomplexan cell division, which involves an assembly in the parental cell, followed by *de novo* synthesis and packaging of the organelles [10,45]. Our analysis revealed that tachyzoites lacking TgPP6C and TgPP6R exhibit replication defects, characterized by asynchronous division (RHΔ*pp6c* and RHΔ*pp6r*) and formation of one or more than two daughter buds within the parental parasite (RHΔ*pp6c*), suggesting that TgPP6C and TgPP6R contribute to tachyzoite proliferation, particularly through their role in endodyogeny.

The centrosomes and microtubule organizing centers (MTOCs) play an indispensable role in *T*. *gondii* morphology, structure, coordinating daughter cells formation, and organelle biogenesis [4,46–48]. In our study, colocalization with the centrosome marker TgCentrin1 showed that centrosome stability was affected by deletion of TgPP6C, with a marked increase in endopolygeny events in the mutant strain. The centrosome inner core contains TgCEP250 and TgCEP250L1 proteins, and is tightly aligned with the centrocone, a mitotic structure embedded in the nuclear envelope and mediates the connection between kinetochores and the centrosome inner core [4,46]. However, TgPP6C was not involved in the centromere function, even though its absence resulted in nuclear segregation defects. Centrosome duplication is followed by a process of budding, consequently, the increase in endopolygeny within the RHΔ*pp6c* strain is likely attributed to instability of centrosomes.

The family of PPPs has evolved to exploit the promiscuous nature of the catalytic subunits and their own serine/threonine specificity to confer substrate specificity via specific interactions with numerous regulatory subunits [15,49]. *T*. *gondii* PPPs share the same core catalytic domain as most eukaryotes, and TgPP6C possesses 6 motifs GDIHG, GDYVDRG, GNHE, HGG, RG and H. Thus, we generated six TgPP6C mutants, each with a mutation in one core motif. Disruption of the individual motifs via mutation to alanine, showed that these motifs have an indispensable role in the biological functions of TgPP6C, although they have no or minimal impact on the localization of TgPP6C.

Phosphorylation is a post-translational modification that regulates the function of proteins, which mediate many molecular processes, such as apoptosis, cell cycle and signal transduction [15,50–53]. The differential regulation of phosphoproteins during *T*. *gondii* infection has been studied using proteomic approaches [54]. In the present study, we used a label-free quantitative phosphoproteomic analysis to identify alterations in protein phosphorylation status associated with deletion of TgPP6C, such as those involved in *T*. *gondii* cell cycle. For example, TgLIPIN is a phosphatidic acid phosphatase (PAP), localizes to the cytosolic-ER interface [33], was significantly phosphorylated at multiple sites when TgPP6C was deleted. The inducible disruption of TgLIPIN leads to membrane malformation, mostly at the IMC and endomembrane system, causing synchronous division and replication defects [33].

In addition, TgPP6C deletion caused differential phosphorylation status of numerous IMCs and IMC component proteins. These are involved in various stages of the division cycle of tachyzoites, where IMC10, IMC14, IMC29 and ISP2 cause division defects during endodyogeny, whereas GAP40, GAPM2B and GAPM3 play roles in maintaining IMC stability during parasite replication [32,38,39,41,42]. Interestingly, *T*. *gondii* PPP family member TgPP1 contributes to the assembly of IMC within the daughter cells, where its deletion leads to differential phosphorylation status of IMC proteins [55].

The phosphorylation status of specific daughter scaffold components may be critical for their accurate assembly. Insufficient availability of these building blocks may lead to the formation of multiple daughter buds, instigating a subsequent cycle of centrosome duplication, and thus increasing the incidence of endopolygeny [42]. Hence, it is reasonable to assume that deletion of TgPP6C alters the phosphorylation status of multiple IMCs and IMC component proteins. Among these, certain proteins may be recruited at the earliest stage of budding and function as structural elements in the emerging daughter cells. For example, IMC29 is one of the earliest proteins expressed during endodyogeny which is essential *in vitro* and *in vivo* in type I RH strain [32]. The vacuoles of Δ*imc29* exhibit a high rate of uncoordinated division, resulting in daughter buds at various stages of maturation in the vacuoles, which is somewhat similar to what was observed in RHΔ*pp6c* [32]. Notably, after TgPP6C deletion, the phosphoprotein IMC29, which contains two differentially phosphorylated sites (S1106 and T891) was upregulated. The phosphorylation site S1106 is dispensabile for the localization and function of IMC29, whereas the localization and function of the phosphorylation site T891 remains to be elucidated [32].

On the other hand, 427 phosphosites on 213 proteins (encompassing most of the differentially abundant phosphoproteins involved in the cell cycle) showed reduced phosphorylation after deletion of TgPP6C. We attribute this finding to possible secondary effects related to activation of other protein phosphatases or inhibition of kinases caused by TgPP6C deletion.

Both TgPP6C and TgPP6R are important for *T*. *gondii* virulence, but only RHΔ*pp6c* exhibited promising properties as a live-attenuated vaccine for preventing toxoplasmosis. Given its significantly defective growth and abnormal divisions observed *in vitro*, we hypothesized that RHΔ*pp6c* may exhibit attenuated virulence *in vivo*. Mice infected with 100 or 200 tachyzoites of RHΔ*pp6c* did not exhibit toxoplasmosis symptoms, thus we measured the anti-*T*. *gondii* antibody levels after infection with 200 tachyzoites of RHΔ*pp6c*. Similar to other live-attenuated vaccine candidates, mice immunized with RHΔ*pp6c* showed high levels of IgG antibodies, which activate the classical complement pathway to control *T*. *gondii* infection [56–58]. Subsequent challenge with the WT strains (high dose of type I and ToxoDB#9 tachyzoites and low dose of type II tissue cysts) showed that RHΔ*pp6c* completely protected mice against acute infection, and significantly reduced the formation of brain cysts in chronically infected mice. Further studies are needed to explore protection against other forms of toxoplasmosis, such as ocular or congenital infection.

In summary, this study revealed the important role of TgPP6 in the synchronous replication of *T*. *gondii* tachyzoites. TgPP6C and TgPP6R may form a heterotrimer with TGGT1_203150 but not with TgPP6S. TgPP6C and TgPP6R play key roles in the *in vitro* proliferation and *in vivo* pathogenicity of *T*. *gondii*. In addition, six conserved motifs of TgPP6C were found essential for the function of TgPP6C. Comparative phosphoproteomics revealed deregulation of phosphorylation and significant enrichment of cell cycle associated proteins. Immunization with RHΔ*pp6c* induced a high level of Th1/Th2 response and conferred a significant protection to the mice from a subsequent challenge by the lethal dose of type I and type ToxoDB#9 strains, and low dose of type II tissue cysts, suggesting that TgPP6C is a relevant candidate for the development of a live attenuated vaccine to control toxoplasmosis.

## Materials and methods

### Ethics statement

The study protocol was reviewed and approved by the Research Ethics Committee of Lanzhou Veterinary Research Institute, Chinese Acdemy of Agricultural Sciences (Permit No. 2021–006). All experiments were carried out in accordance with the national guidelines.

### Animals

Seven-to-eight week old Kunming mice were housed in environmentally enriched conditions with 12-h light/dark cycle. Mice were acclimatized to the local environment for at least one week prior to the experiment [17, 59]. Unless otherwise stated, six mice were used per group.

### Cell line and parasite growth conditions

Tachyzoites of *T*. *gondii* type I RHΔ*ku80* (referred to as WT), RHΔ*ku80*::TIR1 (RH::TIR1) and ToxoDB#9 (PYS) strains were maintained in monolayers of human foreskin fibroblasts (HFFs, ATCC, Manassas, VA, USA), as previously described [17,59]. HFFs were maintained in Dulbecco’s modified Eagle’s medium (DMEM) supplemented with 10% fetal bovine serum (FBS) and incubated at 37 °C with 5% CO_2_. Cysts of *T*. *gondii* type II Pru strain were obtained from the brain homogenates of Kunming mice as previously described [56,59].

### Construction of the deletion strains and epitope tagging

Knock-out and epitope tagged strains were constructed using the clustered regularly interspaced short palindromic repeats (CRISPR)-Cas9 system as previously described [17,59]. All the primers used are listed in S5 Table. For the CRISPR plasmid of each knock-out strain, pSAG1:CAS9-U6:sgUPRT was used as a template, and the corresponding sgRNA of each gene was replaced with sgUPRT, as previously described [59]. For the construction of 5HR-DHFR-3HR, according to the Gibson assembly kit protocol (New England Biolabs, USA), the 5’, 3’ homologous fragments and DHFR of the gene of interest (GOI) were amplified using *T*. *gondii* genomic DNA and pUPRT-DHFR-D plasmid, respectively. The three fragments were ligated to replace the coding region of GOI, as previously described [17,59]. For the construction of GOI epitope-tagged strain, using pLIC-6×HA-DHFR as the template, a fragment of about 42 bp containing the GOI gene and a tagged product containing 6×HA were amplified, as previously described [59]. Finally, the specific CRISPR plasmids (~40 μg) and the corresponding fragments (~15 μg) were co-transfected into freshly egressed WT tachyzoites. The transfectants were selected by 3 μM pyrimethamine and isolated via limiting dilution.

### Construction of conditional knock-down strain

To construct conditional knock-down strains, mini-auxin-induced degradation system combined with CRISPR-Cas9 system were used as previously described [60]. Briefly, freshly egressed RH::TIR1 tachyzoite were co-transfected with ~15 μg amplicons flanked with short homology regions containing mAID-6HA and a DHFR maker, and ~40 μg CRISPR plasmids to tag the endogenous genomic locus of GOI at the C-terminus, and the transfectants were selected by limited dilution and grown in medium containing 3 μM pyrimethamine. All the primers used are listed in S5 Table. For knock-down, a final concentration of 500 μM indole-3-acetic acid (IAA) was added to the culture, while 0.1% ethanol was added to the culture for mock treatment.

### Construction of the complemented and mutant strains

For the complete fragment complemented with TgPP6C and the six core motifs specific to the mutated TgPP6C, the complete TgPP6C coding region and the TgPP6C coding region of each core motif (GDIHG, GDYVDRG, GNHE, HGG, RG and H) mutated to alanine were amplified respectively for the construction of pTUB::PP6C::CAT and pTUB::MuPP6C::CAT plasmids, as previously described [17]. All the primers used are listed in S5 Table. About 40 μg pSAG1::Cas9-U6::sgUPRT plasmid and ~15 μg positive complementary or mutant fragment were co-transfected into fresh egressed RHΔ*pp6c* tachyzoites. The transfectants were isolated by limited dilution and grown in medium containing 20 μM chloramphenicol and verified by immunofluorescence staining.

### Immunofluorescence staining and Western blotting

For immunofluorescence, confluent HFF monolayers grown on coverslips were infected by tachyzoites of WT, TgPP6C::6HA, TgPP6S::6HA, TgPP6R::6HA, TGGT1_203150::6HA, RHΔ*pp6c*, RHΔ*pp6c-cp*, RHΔ*pp6r*, RHΔ*pp6r-cp* and PP6C mutants (MU-GDIHG, MU-GDYVDRG, MU-GNHE, MU-HGG, MU-RG and MU-H), and knock-down strains PP6C-mAID-HA and PP6R-mAID-HA with or without IAA, then incubated for 24–36 h. The infected HFFs were washed 3 times with PBS and fixed with 4% paraformaldehyde (PFA) for 20 min. Cells were permeabilized using 0.1% Triton X-100, blocked with 3% bovine serum albumin at room temperature for 30 min, and incubated with primary antibodies overnight at 4°C. The primary antibodies used in this study included mouse anti-SAG1 (1:1000), rabbit anti-IMC1 (1:500), mouse anti-IMC1 (1:500), rabbit anti-SERCA (1:500), rabbit anti-HA (1:1000), rabbit anti-Chromo1 (1:500), rabbit anti-Centrin1 (1:500), rabbit anti-GRA32 (1:500), rabbit anti-ISP1 (1:500), rabbit anti-ARO (1:500), rabbit anti-CPN60 (1:500), rabbit anti-HSP60 (1:500), rabbit anti-MIC2 (1:500) and rabbit anti-GAP45 (1:500) (prepared in our laboratory). The secondary antibodies conjugated with Alexa Fluor 488/594 (Invitrogen, USA) were added at appropriate dilutions in PBS for 1 h at 37 °C in the dark. The cell nuclei were counter-stained with DAPI (4’,6-diamidino-2-phenylindole), and the cells were photographed under a Leica confocal microscope (TCS SP8, Leica, Germany).

For Western blot analysis, freshly egressed tachyzoites of the WT, TgPP6C::6HA, TgPP6S::6HA and TgPP6R::6HA strains were collected and lysed on ice using RIPA lysis buffer (Thermo Fisher Scientific, USA). The target protein was analyzed by SDS-PAGE and transferred to PVDF membrane (Immobilon, Millipore, USA). The Western blotting antibodies used in this study were rabbit anti-aldolase (1:500) and rabbit anti-HA (1:1000). Images were acquired on a Minichemi 610 chemiluminescence imager (Sagecreation, Beijing, China), as previously described [61].

### Phenotypic characterization of the mutant strains

The standard plaque formation assay was performed as previously described [59]. Freshly egressed tachyzoites of the WT and mutant strains were collected, and diluted to 10^3^/mL or 2.5 × 10^3^/mL. According to the experimental requirements, and selecting the appropriate concentration, 200 μL of tachyzoites of the WT and mutant strains were added to monolayers of HFFs grown on 12-well tissue culture plastic plates. For conditional knock-down, 500 μM IAA or 0.1% ethanol was added to the medium before the addition of RH::TIR1 or mutant tachyzoites. The plates were incubated at 37 °C with 5% CO_2_ for 7 days before fixation and staining with 0.5% crystal violet. The size and number of plaques were determined using a scanner as previously described [59]. All plaque assays were performed in triplicate.

The ability of tachyzoites of WT, RHΔ*pp6c*, RHΔ*pp6c-cp*, RHΔ*pp6r* and RHΔ*pp6r-cp* to invade host cells was determined by the invasion assay, as previously described [59]. Briefly, tachyzoites of WT, RHΔ*pp6c* and RHΔ*pp6c-cp* were harvested, counted, and diluted to a final concentration of 2 × 10^7^/mL. Then, 100 μL was added to coverslips positioned in the bottom of 12-well culture plates with confluent HFF monolayers and incubated for 40 min, followed by washing to remove the unattached tachyzoites. As for the knock-down and RH::TIR1 strains, tachyzoites were pretreated with 500 μM IAA or 0.1% ethanol for 16 h before being added to the well. The cells were fixed with 4% PFA. The mouse anti-SAG1 and Alexa Fluor 488 goat anti-mouse antibodies were added, followed by washing with PBS to remove unbound antibodies. Before the second round of immunolabeling, cells were permeabilized with 0.1% Triton X-100 in PBS. Then secondary antibodies mouse anti-SAG1 and Alexa Fluor 594 goat anti-mouse were added. The ratio of intracellular/total tachyzoites was used to determine parasite invasion efficiency, as previously described [59]. At least 20 random microscopic fields from three separate experiments per sample were examined.

For the intracellular replication assay, tachyzoites of WT, RHΔ*pp6c*, RHΔ*pp6c-cp*, RHΔ*pp6r* and RHΔ*pp6r-cp* were adjusted to a final concentration of 1 × 10^6^/mL. Then, 100 μL of tachyzoite suspension of each strain was inoculated into confluent HFFs grown in 6-well culture plates for 1 h. The tachyzoites remained attached to the cell surface were washed with PBS and the plates were further incubated at 37 °C with 5% CO_2_ for 31 h. For knock-down and RH::TIR1 strains, parasites were pretreated with 500 μM IAA or 0.1% ethanol for 16 h before being used in the replication assay. The cells were fixed with 4% PFA, followed by addition of mouse anti-SAG1 and Alexa Fluor 488 goat-anti mouse as primary and secondary antibodies, respectively. For each strain, at least 200 PVs per well were counted to determine the number of PVs enclosing 1–3, 4–7, 8–15, 16–32 and >32 tachyzoites, and the number of abnormal PVs with asynchronous division was recorded. Replication assay data were obtained from three independent experiments.

For the egress assay, tachyzoites of WT, RHΔ*pp6c*, RHΔ*pp6c-cp*, RHΔ*pp6r* and RHΔ*pp6r-c* were adjusted to a final concentration of 1 × 10^6^/mL. Then, 100 μL of tachyzoite suspension of each strain was inoculated into 12 well culture plates containing monolayers of HFFs for 1 h. After washing the unbound tachyzoites and continued incubation for 36–48 h, tachyzoites were treated with DMEM (pre-warmed at 37 °C) containing 3 μM calcium ionophore A23187. For the knock-down and RH::TIR1, tachyzoites were grown in culture media containing 500 μM IAA or 0.1% ethanol for 36–48 h before the induction of egress. After treatment with calcium ionophore for 2 min, the cells were immediately fixed. For each strain, at least 200 PVs per well were counted to determine the number of PVs with egressed tachyzoites as a percentage of the total number of PVs [59]. Egress assays were performed three separate times.

For transmission electron microscopy, HFF monolayers were infected with WT and RHΔ*pp6c* strains for 20 and 30 h, respectively, and fixed with 2.5% glutaraldehyde in 0.1 M sodium cacodylate buffer (pH 7.4) for 2 h at room temperature. The samples were washed in cacodylate buffer, post-fixed in 1% osmium tetroxide, and processed as previously described [61]. Images were captured using a HITACHI HT7700 electron microscope under 80 kV. The data are obtained from three independent experiments.

### Virulence assessment in mice

To assess the role of TgPP6 in the virulence of *T*. *gondii*, groups (*n* = 6) of Kunming mice were injected with WT strain or mutant strains (RHΔ*pp6c*, RHΔ*pp6c-cp*, RHΔ*pp6r* and RHΔ*pp6r-cp*) intraperitoneally (i.p.) at an inoculum of 100 tachyzoites in a volume of 200 μL sterile PBS. Another group of mice was inoculated i.p. with 200 μL PBS only and served as a control group (naïve). The infected mice were monitored twice daily for signs suggestive of *T*. *gondii* infection for 30 days. Mice were euthanized once they reach their humane endpoint (e.g., body weight loss of ≥20%). The virulence of the different strains was compared using the survival curve of the mice. For immunization of mice using RHΔ*pp6c*, the mice were inoculated i.p. with 200 μL PBS containing 200 RHΔ*pp6c* tachyzoites or mock-immunized (naïve) with 200 μL PBS. To examine the immune responses induced by immunization using RHΔ*pp6*c, enzyme-linked immunosorbent assay (ELISA) was used for quantification of anti-*T*. *gondii* total IgG and subclasses of IgG (IgG1, and IgG2a) antibodies 30 days after immunization, as previously described [56]. To assess the protective efficacy of immunization against acute infection, RHΔ*pp6c* immunized and naïve mice (6 mice/group) were injected i.p. with a lethal challenge of type I strain RH or ToxoDB#9 strain PYS (10^3^ tachyzoites /mouse) in a volume of 200 μL PBS, 30 days after immunization. We also evaluated the efficacy of protection against chronic *T*. *gondii*, where immunized mice and naïve mice were gavaged orally with 20 cysts of type II Pru strain. All challenged mice were observed twice daily for morbidity (hunched posture, ruffled fur inactivity, body weight loss) to 30 d after re-challenge. The survived mice were euthanized to determine their brain cyst burden by homogenizing their brains, 1 brain in 1 mL of PBS, as previously described [56,59].

### Immunoprecipitation assay

Immunoprecipitation assay was performed using the Pierce Magnetic HA-Tag IP/Co-IP kit (ThermoFisher Scientific, USA), as previously described [17]. In brief, the mixture of 50 μL of anti-HA beads and the supernatant of parasite lysates was incubated for 3 h at 4 °C under end-to-end rotation. Protein was obtained by adding 100 μL of elution buffer. The eluted proteins were separated by SDS-PAGE. After colloidal Coomassie staining, eight horizontal gel slices were excised in strip lanes and the proteins were processed with trypsin (Promega/Pierce) using a Janus liquid handling system (PerkinElmer) in-gel digestion. Subsequently, peptides were analyzed by liquid chromatography-mass spectrometry (LC-MS), the RAW data were searched against the *T*. *gondii* databases in ToxoDB (https://toxodb.org). Peptides and proteins were identified with a 1% false discovery rate (FDR), and proteins with single modification sites, contaminants, and reversed proteins were all removed.

### Phosphoproteomic analysis

Tachyzoites of the WT and RHΔ*pp6c* were used to infect T-175 cell culture plastic flasks containing monolayers of HFFs using a multiplicity of infection (MOI) of 5. To compensate for the low rate of cell division in mutant strain, RHΔ*pp6c* was cultured for a longer duration compared to wild type. Therefore, cells infected by WT or RHΔ*pp6c* tachyzoites were incubated for 36 h or 48 h, respectively, to maximize the number of tachyzoites inside the PVs, followed by washing with PBS and storage at –80 °C. For protein extraction, lysis buffer (1% SDS, 1% protease inhibitor cocktail, 1% phosphatase inhibitor for phosphorylation) was added to each sample and vortexed for 5 min. The mixture was sonicated in an ice bath (sonication for 3 s, stop for 5 s, 25% power, power 220 W) and lysed for 3 min, then centrifuged at 12,000 *g* for 10 min at 4 °C. The supernatant was collected and protein concentration of each sample was determined by BCA method.

For trypsin digestion and LC-MS/MS analysis, 30 μg of the protein solution was used for electrophoresis, followed by enzymatic digestion of the same amount of protein, and the volume was adjusted to the same volume with lysis buffer. Then, 1 volume of pre-cooled acetone was added, followed by vortexing and addition of 4 times of acetone at –20 °C/2 h. After centrifugation at 4500 *g* for 5 min, acetone was added for washing. After the precipitate was air-dried, triethylammonium bicarbonate was added, and the ultrasound machine was used to break up the precipitate. Trypsin was added at 1:50 protease-to-protein mass ratio and the enzyme was hydrolyzed overnight. Dithiothreitol was added to a final concentration of 5 mM at 56 °C for 30 min. Then, iodoacetamide was added to make a final concentration of 11 mM, followed by incubation at room temperature for 15 min in the dark. Finally, the peptides were desalted by Strata X SPE column. The peptides were dissolved in loading buffer solution (50% acetonitrile/0.5% acetic acid), the supernatant was transferred to IMAC microspheres suspension and incubated on a rotary shaker with gentle shaking. To remove non-specifically adsorbed peptides and elute the enriched phosphopeptides, IMAC microspheres were washed sequentially with 50% acetonitrile/0.5% acetic acid and 30% acetonitrile/0.1% trifluoroacetic acid, and then an elution buffer containing 10% NH_4_OH was added.

The tryptic peptides were dissolved in solvent A, loaded onto a home-made reversed-phase analytical column (25-cm length, 75 μm i.d.). Solvent A was an aqueous solution containing 0.1% formic acid and 2% acetonitrile, and solvent B was a solution containing 0.1% formic acid and 100% acetonitrile. The gradient settings were 0–70 min 6%~24%B, 70–84 min 24%~35%B, 84–87 min 35%~80%B and 87–90 min 80%B, and the flow rate was maintained at 500nL/min on an EASY-nLC 1200 system. The peptides were subjected to capillary ion source for analsyis using TimsTOF Pro mass spectrometer (Bruker Daltonics). The electrospray voltage and scan range were 1.60 kV and 100–1700 m/z, respectively.

For bioinformatics analysis, the resulting MS/MS spectra were searched against *T*. *gondii* GT1 database ToxoDB (https://toxodb.org) using MaxQuant search engine (v.1.6.15.0). Peptide and protein identifications were filtered to a 1% false discovery rate (FDR). Fold-Change (FC) > 1.5 or < –1.5 and *P* < 0.05 were set as the threshold for differentially phosphorylated proteins. The significantly enriched gene ontology (GO) terms of the differentially phosphorylated proteins were identified by comparing to the parasite genome in ToxoDB database (https://toxodb.org).

### Statistical analysis

All statistical analyses were performed using GraphPad Prism software version 8.4.0 for MacOS (GraphPad, CA, USA) using two tailed unpaired Student’s *t*-test and two-way analysis of variance (ANOVA). Survival curves were compared using the log-rank (Mantel-Cox) test. Data are based on three independent biological replicates unless otherwise specified. *P* values < 0.05 were considered significant.

## Supporting information

S1 Fig Epitope tagging of TgPP6 holoenzyme in *Toxoplasma gondii*. (**A**) Schematic showing endogenous tagging of the gene of interest (GOI) at the C-terminus, and the mAID system used to conditionally inactivate GOI, dihydrofolate reductase (DHFR) resistance cassette for selection using pyrimethamine. (**B**) PCR1 and PCR2 showed the correct integration of tags in the indicated strains. (**C**) Western blotting detected the expression of the hemagglutinin (HA)-tagged TgPP6 in RH tachyzoites. The bands of 6×HA tagged TgPP6C, TgPP6R and TgPP6S were detected at approximately 97, 121 and 45 kDa, respectively.
(TIF)

S2 Fig Construction of the TgPP6 subunits knock-out and complemented strain. (**A**) Schematic illustration shows the targeted deletion of the coding region of TgPP6. (**B**) Diagnostic PCR3 and PCR5 confirmed the correct 5’ and 3’ region integration of homologous fragments, respectively, and PCR4 confirmed the deletion of TgPP6 coding region. (**C**) Complementation strategy of TgPP6C and TgPP6R into UPRT locus and isolation of clones by chloramphenicol. (**D**) and (**E**) The invasion efficiency of RHΔ*pp6c* and RHΔ*pp6r* strains was slightly lower than that of WT, RHΔ*pp6c-cp* and RHΔ*pp6r-cp* strains, while the egress rate of RHΔ*pp6c* and RHΔ*pp6r* strains was similar to the other strains. Data represent mean ± SD of three independent experiments and significance was analyzed by unpaired *t*-test. *P = 0.0396 (RHΔ*pp6c* vs. WT); *P = 0.0448 (RHΔ*pp6r* vs. WT); n.s., not significant.
(TIF)

S3 Fig Conditional depletion of TgPP6C and TgPP6R produces a phenotype similar to that observed in RHΔ*pp6c* and RHΔ*pp6r*. (**A**) Plaque assay of the indicated strains treated with or without IAA for 7 days. The arrows indicate plaque formed by PP6C-mAID and PP6R-mAID with IAA. (**B**) The relative size of the plaques for each strain. Data represent mean ± SD of three independent experiments and statistical differences were analyzed by the unpaired *t*-test. ****P < 0.0001; n.s., not significant. (**C**) and (**D**) Immunofluorescence staining shows PP6C-mAID (red) and PP6R-mAID (red) strains, cultured with or without IAA for 24 h, stained against TgIMC1 (green). Knock-down of TgPP6C and TgPP6R resulted in asynchronous division of tachyzoites. Scale bars: 2 μm. (**E**) Replication of PP6C-mAID and PP6R-mAID parasites after growth in the presence or absence of IAA for 32 h. Data are mean ± SD from three independent experiments, > 200 vacuoles were counted per replicate and were analyzed by a two-way ANOVA; *P = 0.0178; ***P = 0.0001; ****P < 0.0001; n.s., not significant. (**F**) and (**G**) Invasion and egress assays showed that PP6C-mAID and PP6R-mAID pretreated with IAA slightly affected parasite invasion, but not egress. Data represent mean ± SD and The three independent data were analyzed for statistical significance by the unpaired *t*-test. *P = 0.0237 (PP6C-mAID +IAA vs. PP6C-mAID–IAA); *P = 0.0232 (PP6R-mAID +IAA vs. PP6R-mAID–IAA); n.s., not significant.
(TIF)

S4 Fig TgPP6C deletion has no notable effects on the structure of *T*. *gondii* rhoptries, dense granules, apicoplast, mitochondria or micronemes. IMC1 (green) and DAPI (blue) stains were used to denote the parasites and nuclei (DNA), respectively. Anti-ARO, anti-GRA32, anti-CPN60, anti-HSP60 and anti-MIC2 antibodies were used to stain the rhoptries, dense granules, apicoplast, mitochondria and micronemes, respectively. Scale bars: 2 μm.
(TIF)

S1 Table Summary of the mass spectrometry analysis of *T*. *gondii* proteins immunoprecipitated with TgPP6C::6HA, TgPP6R::6HA and TgPP6S::6HA. (XLSX)

S2 Table Summary of the proteomic analysis of RHΔ*pp6c* vs WT strain. (XLSX)

S3 Table Summary of the phosphoproteomic analysis of RHΔ*pp6c* vs WT strain. (XLSX)

S4 Table Quantitative data of *T*. *gondii* phosphopeptides of the differentially phosphorylated proteins of RHΔ*pp6c* vs WT strain. (XLSX)

S5 Table Primers used in the construction of the knock-out, epitope-tagged, complemented and mutant strains. (DOC)
