# Supplementary figures and images for: The Toxoplasma protein phosphatase 6 catalytic subunit (TgPP6C) is essential for cell cycle progression and virulence
Source: PLoS Pathog. 2023 Dec 13;19(12):e1011831. doi: 10.1371/journal.ppat.1011831 (PMC10752510; doi:10.1371/journal.ppat.1011831)

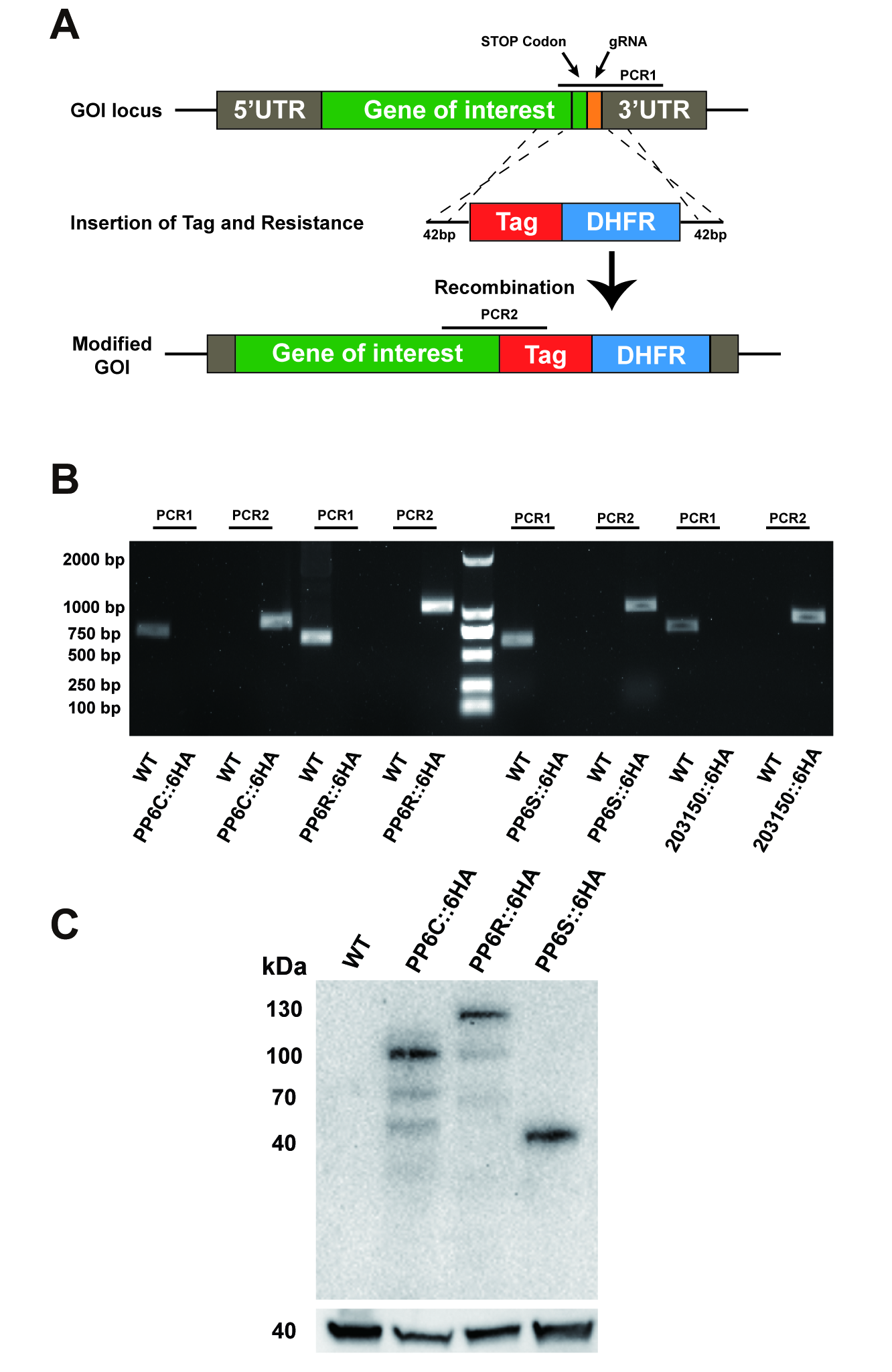

Supplement: S1 Fig — (A) Schematic showing endogenous tagging of the gene of interest (GOI) at the C-terminus, and the mAID system used to conditionally inactivate GOI, dihydrofolate reductase (DHFR) resistance cassette for selection using pyrimethamine. (B) PCR1 and PCR2 showed the correct integration of tags in the indicated strains. (C) Western blotting detected the expression of the hemagglutinin (HA)-tagged TgPP6 in RH tachyzoites. The bands of 6×HA tagged TgPP6C, TgPP6R and TgPP6S were detected at approximately 97, 121 and 45 kDa, respectively. (TIF) [file ppat.1011831.s001.tif]

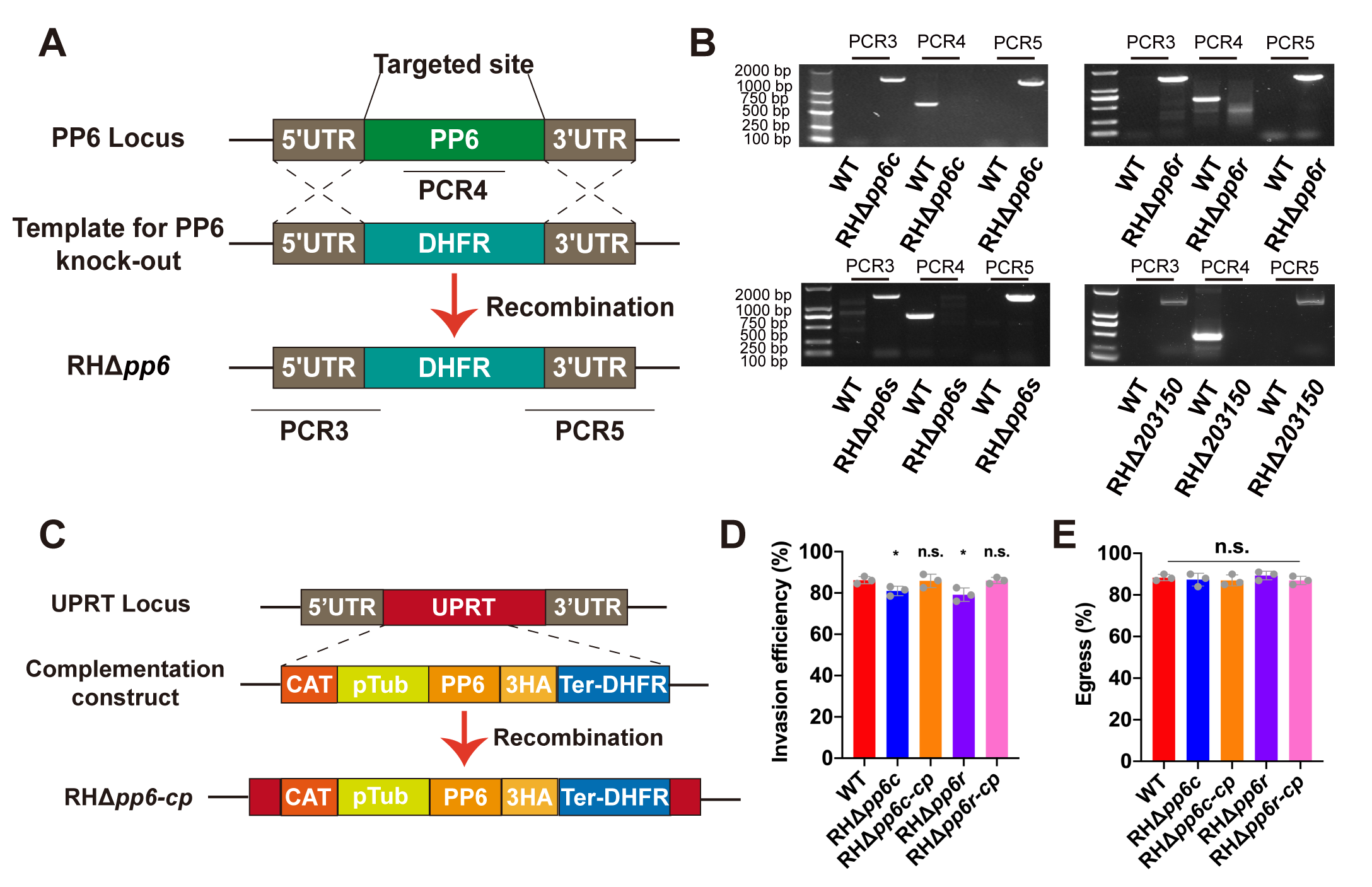

Supplement: S2 Fig — (A) Schematic illustration shows the targeted deletion of the coding region of TgPP6. (B) Diagnostic PCR3 and PCR5 confirmed the correct 5’ and 3’ region integration of homologous fragments, respectively, and PCR4 confirmed the deletion of TgPP6 coding region. (C) Complementation strategy of TgPP6C and TgPP6R into UPRT locus and isolation of clones by chloramphenicol. (D) and (E) The invasion efficiency of RHΔpp6c and RHΔpp6r strains was slightly lower than that of WT, RHΔpp6c-cp and RHΔpp6r-cp strains, while the egress rate of RHΔpp6c and RHΔpp6r strains was similar to the other strains. Data represent mean ± SD of three independent experiments and significance was analyzed by unpaired t-test. *P = 0.0396 (RHΔpp6c vs. WT); *P = 0.0448 (RHΔpp6r vs. WT); n.s., not significant. (TIF) [file ppat.1011831.s002.tif]

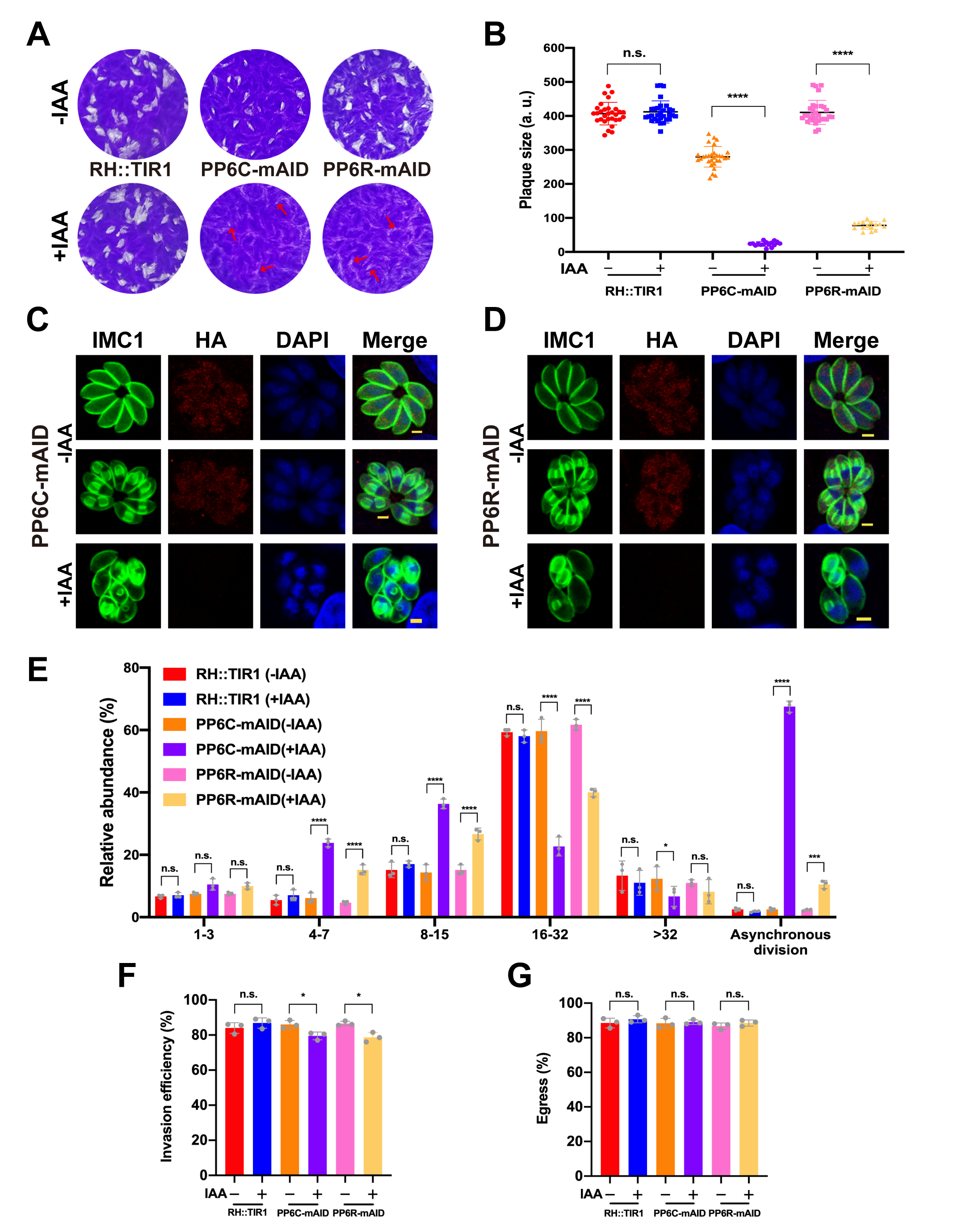

Supplement: S3 Fig — (A) Plaque assay of the indicated strains treated with or without IAA for 7 days. The arrows indicate plaque formed by PP6C-mAID and PP6R-mAID with IAA. (B) The relative size of the plaques for each strain. Data represent mean ± SD of three independent experiments and statistical differences were analyzed by the unpaired t-test. ****P < 0.0001; n.s., not significant. (C) and (D) Immunofluorescence staining shows PP6C-mAID (red) and PP6R-mAID (red) strains, cultured with or without IAA for 24 h, stained against TgIMC1 (green). Knock-down of TgPP6C and TgPP6R resulted in asynchronous division of tachyzoites. Scale bars: 2 μm. (E) Replication of PP6C-mAID and PP6R-mAID parasites after growth in the presence or absence of IAA for 32 h. Data are mean ± SD from three independent experiments, > 200 vacuoles were counted per replicate and were analyzed by a two-way ANOVA; *P = 0.0178; ***P = 0.0001; ****P < 0.0001; n.s., not significant. (F) and (G) Invasion and egress assays showed that PP6C-mAID and PP6R-mAID pretreated with IAA slightly affected parasite invasion, but not egress. Data represent mean ± SD and The three independent data were analyzed for statistical significance by the unpaired t-test. *P = 0.0237 (PP6C-mAID +IAA vs. PP6C-mAID–IAA); *P = 0.0232 (PP6R-mAID +IAA vs. PP6R-mAID–IAA); n.s., not significant. (TIF) [file ppat.1011831.s003.tif]

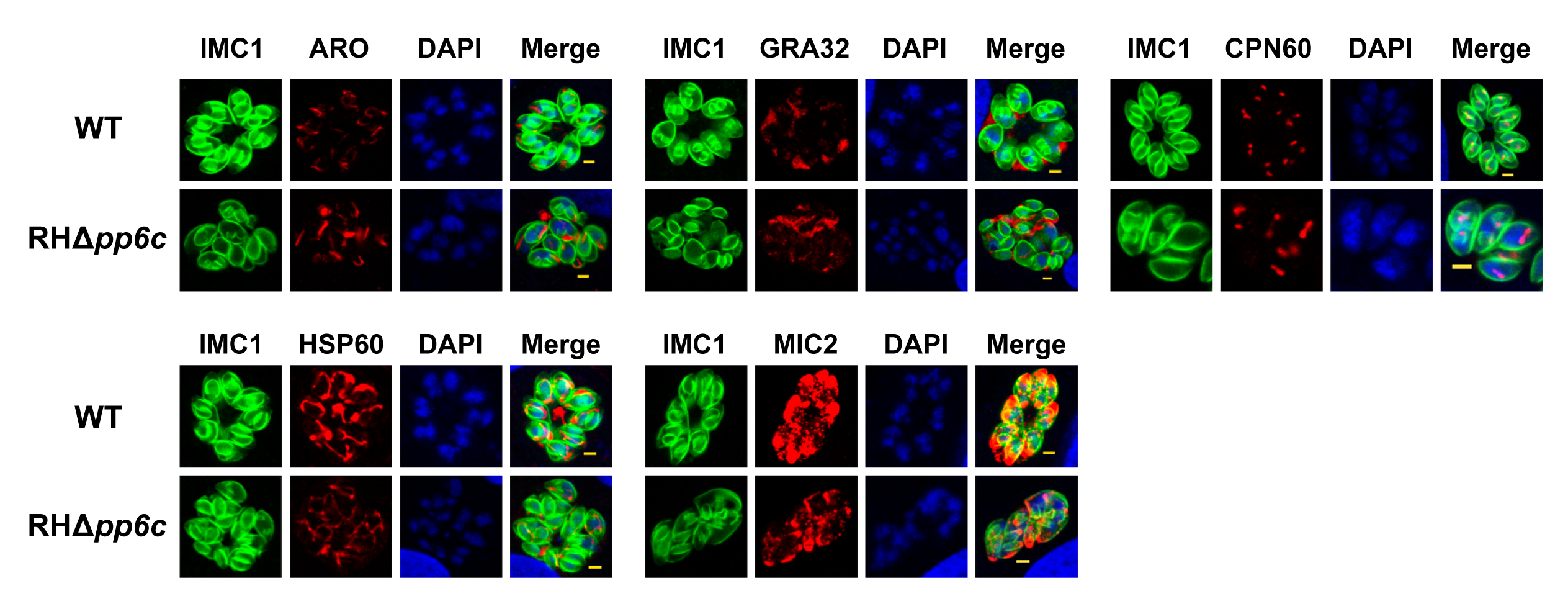

Supplement: S4 Fig — IMC1 (green) and DAPI (blue) stains were used to denote the parasites and nuclei (DNA), respectively. Anti-ARO, anti-GRA32, anti-CPN60, anti-HSP60 and anti-MIC2 antibodies were used to stain the rhoptries, dense granules, apicoplast, mitochondria and micronemes, respectively. Scale bars: 2 μm. (TIF) [file ppat.1011831.s004.tif]
