# Supplementary material for: The Toxoplasma protein phosphatase 6 catalytic subunit (TgPP6C) is essential for cell cycle progression and virulence
Source: PLoS Pathog. 2023 Dec 13;19(12):e1011831. doi: 10.1371/journal.ppat.1011831 (PMC10752510; doi:10.1371/journal.ppat.1011831)
Supplement: S5 Table — (DOC) [file ppat.1011831.s009.doc]

**S****5 Table. Primers used in the construction of knock-out, epitope-tagged, complemented and mutant strains.**

| **Primer** | **Sequence (5**′**——3**′**)** | **Use** |
| --- | --- | --- |
| SgPP6C-F | GGAGTGAGAAGAGCTGTCTCGTTTTAGAGCTAGAAATAGC | To construct the TgPP6C‐specific CRISPR plasmid for gene deletion |
| SgPP6R-F | GCGAAAGACGCGTCTTCCCCGTTTTAGAGCTAGAAATAGC | To construct the TgPP6R‐specific CRISPR plasmid for gene deletion |
| SgPP6S-F | GATGAACGATCTGGACCTCCGTTTTAGAGCTAGAAATAGC | To construct the TgPP6S‐specific CRISPR plasmid for gene deletion |
| Sg203150-F | GAAGAGGCGGAGCTCGACCCGTTTTAGAGCTAGAAATAGC | To construct the Tg203150‐specific CRISPR plasmid for gene deletion |
| 3′SgPP6C-F | GCTGTATCTCAGAGAAAGTAGTTTTAGAGCTAGAAATAGC | To construct the 3′TgPP6C‐specific CRISPR plasmid for C-terminal tagging |
| 3′SgPP6R-F | TTCTTAGGAACAAAGACCTGGTTTTAGAGCTAGAAATAGC | To construct the 3′TgPP6R‐specific CRISPR plasmid for C-terminal tagging |
| 3′SgPP6S-F | TTTCTTGTTAACGTCTCTTCGTTTTAGAGCTAGAAATAGC | To construct the 3′TgPP6S‐specific CRISPR plasmid for C-terminal tagging |
| 3′Sg203150-F | GGCTGTCTTTCCGTCCTCGTGTTTTAGAGCTAGAAATAGC | To construct the 3′Tg203150‐specific CRISPR plasmid for C-terminal tagging |
| gRNA-R | AACTTGACATCCCCATTTAC | To construct gene‐specific CRISPR plasmids |
| U5PP6C‐F | GGTTTTCCCAGTCACGACGTTCTTCTTCCGTCTGCTTCG | Amplification of 5′‐homology fragment of PP6C |
| U5PP6C‐R | GGATTTACAGCCTGGCGAAGCTTGCCTTCTTGTCGAGAGTG |  |
| U5PP6R‐F | GGTTTTCCCAGTCACGACGTTTGTTGGAAGAGGAGGAAGA | Amplification of 5′‐homology fragment of PP6R |
| U5PP6R‐R | GGATTTACAGCCTGGCGAAGCTTCATTTGTGTTGGCGAGAAA |  |
| U5PP6S-F | GGTTTTCCCAGTCACGACGTTTCTCTTCTCCTTCCTCCTC | Amplification of 5′‐homology fragment of PP6S |
| U5PP6S-R | GGATTTACAGCCTGGCGAAGCTTCAAGTCAAGAATGCTCGTAA |  |
| U5203150-F | GGTTTTCCCAGTCACGACGTTGGTTGTTCCACTCGTCTG | Amplification of 5′‐homology fragment of 203150 |
| U5203150-R | GGATTTACAGCCTGGCGAAGCTTGAAGAAGGAGAAGCCGTAG |  |
| U3PP6C‐F | CTATGCACTTGCAGGATGAATTCTGGAGACAAAGTGGTGAAG | Amplification of 3′‐homology fragment of PP6C |
| U3PP6C‐R | GAGCGGATAACAATTTCACACCAGCATTGACTCATAGAAC |  |
| U3PP6R-F | CTATGCACTTGCAGGATGAATTCCAGCAGATGATCCTCGTAA | Amplification of 3′‐homology fragment of PP6R |
| U3PP6R-R | GAGCGGATAACAATTTCACAGCCGTGATAAGAAGAGAATG |  |
| U3PP6S-F | CTATGCACTTGCAGGATGAATTCTGGACTACGCTCTGTCTTAC | Amplification of 3′‐homology fragment of PP6S |
| U3PP6S-R | GAGCGGATAACAATTTCACATTATGACGCCGAGGAACC |  |
| U3203150-F | CTATGCACTTGCAGGATGAATTCCACCACTTCTCGCAACTG | Amplification of 3′‐homology fragment of 203150 |
| U3203150-R | GAGCGGATAACAATTTCACACACGGATGGAAGGAGACA |  |
| DHFR-PCR3-R | GCCAAAGTAGAAAGGAATTAGCAT | PCR3 of gene replacement |
| DHFR-PCR5-F | TGACGCAGATGTGCGTGTATCCAC | PCR5 of gene replacement |
| PCR3-PP6C-F | TCTGTTCCATCTCTTCTGCATCT | PCR3 of RHΔ*pp6c* |
| PCR4-PP6C-F | ACATGACACATTCCAAGTTC | PCR4 of RHΔ*pp6c* |
| PCR4-PP6C-R | CTTCAGCAGCATCAAGTAC |  |
| PCR5-PP6C-R | CTTGATTGGAACCACGAAGCAC | PCR5 of RHΔ*pp6c* |
| PCR3-PP6R-F | TCTGTTCTCCTTTCTGATGTTCC | PCR3 of RHΔ*pp6r* |
| PCR4-PP6R-F | ACGAAGGAATGGAGTTGTT | PCR4 of RHΔ*pp6r* |
| PCR4-PP6R-R | GTGTTGAACTGTGATGTTGA |  |
| PCR5-PP6R-R | GTCAGAGTCAGCTAGGAATCAGG | PCR5 of RHΔ*pp6r* |
| PCR3-PP6S-F | TCTGCTTTCTTCTTTCACTTGC | PCR3 of RHΔ*pp6s* |
| PCR4-PP6S-F | AGAGAACTCCGAAGAAGAG | PCR4 of RHΔ*pp6s* |
| PCR4-PP6S-R | GCACAGCCATATTCACAG |  |
| PCR5-PP6S-R | TTTCATACAAGAGCGCACGAAT | PCR5 of RHΔ*pp6s* |
| PCR3-203150-F | TATCACAGAGGGACTCTGCTCA | PCR3 of RHΔ*203150* |
| PCR4-203150-F | ACTTCTTCGTCTGCTCAC | PCR4 of RHΔ*203150* |
| PCR4-203150-R | AGGAGAATGGGAGAGGTG |  |
| PCR5-203150-R | TTCAGTGGATGGTCGTGTACA | PCR5 of RHΔ*203150* |
| DHFR-F | AAGCTTCGCCAGGCTGTAAATCC | Amplification of DHFR for Gibson assembly |
| DHFR-R | GAATTCATCCTGCAAGTGCATAG |  |
| pUC19-F | TGTGAAATTGTTATCCGCTC | Amplification of pUC19 for Gibson assembly |
| pUC19-R | AACGTCGTGACTGGGAAAACC |  |
| PP6C-3-HRF | CAAGTCGCCACCCGCGCGCGGGCCATTGCGCCCTACTTTCTCGCTAGCAAGGGCTCGGG | Amplification of PP6C of homology for C-terminal tagging |
| PP6C-3-HRR | CCTCCTCCCCTCCCCCTACGCATACGCTGTATCTCAGAGAAAATACGACTCACTATAGG |  |
| PP6R-3-HRF | AACGCCGCACCGACCAACCGAGAACTTGAGATTTCTCTTTCTGCTAGCAAGGGCTCGGG | Amplification of PP6R of homology for C-terminal tagging |
| PP6R-3-HRR | TGGAGTCCTGGCGGATCGTGTGAAAGGCCGAGGCCGCCGCAGATACGACTCACTATAGG |  |
| PP6S-3-HRF | GGCCTCGAGCTCTTCATTGCGCGAGACAGCCGGAAGAGACGTGCTAGCAAGGGCTCGGG | Amplification of PP6S of homology for C-terminal tagging |
| PP6S-3-HRR | TTCAGCGATAGATCCAGAGGAAGCTTTTCTTGTTAACGTCTCATACGACTCACTATAGG |  |
| 203150-3-HRF | AACTTCGCTCAAAGCGATGGATGGGCTGTCTTTCCGTCCTCGGCTAGCAAGGGCTCGGG | Amplification of 203150 of homology for C-terminal tagging |
| 203150-3-HRR | ATTTCAGGGTGTCCCTCACTATGAGCTTGACCGGTTCCTACGATACGACTCACTATAGG |  |
| Flag-R | CTATTATACCCGTGTGTTACG | To validate tag insertion (PCR2-R) |
| PP6C-C-F | TGGAGACAAAGTGGTGAAG | To validate tag insertion of PP6C (PCR1-F, PCR2-F) |
| PP6C-C-R | AGACACAACGAAGCCAAA | To validate tag insertion of PP6C (PCR1-R) |
| PP6R-C-F | TCACTTCCTCGTCCTTCC | To validate tag insertion of PP6R (PCR1-F, PCR2-F) |
| PP6R-C-R | CACACCATCCTTCGTCTC | To validate tag insertion of PP6R (PCR1-R) |
| PP6S-C-F | CCTTGTGTCTCATTCGTGAA | To validate tag insertion of PP6S (PCR1-F, PCR2-F) |
| PP6S-C-R | AGTTGGATTCGCTCTTATCG | To validate tag insertion of PP6S (PCR1-R) |
| 203150-C-F | GCCTTCCCAGATTTGTGT | To validate tag insertion of 203150 (PCR1-F, PCR2-F) |
| 203150-C-R | CGATGCTCTCTTCTTTCCTA | To validate tag insertion of 203150 (PCR1-R) |
| pPP6C-F | AGTGGAGGACGGGAATTCGGGCCCGGATTTCGCCGACCAACGACTCAT | To construct the start codon of PP6C |
| pPP6C-R | CTGGTGTCGGACCCGCCAGTGCTG |  |
| pPP6R-F | AGTGGAGGACGGGAATTCGGGCCCTGTCTTCCGTGATTCAACACTCGC | To construct the start codon of PP6R |
| pPP6R-R | ACAACCAATCCGGTCTCTCGTGTA |  |
| PP6CCDS-F | CAGCACTGGCGGGTCCGACACCAGATGGAAGAAGGTAATTCCGTGACC | To construct the CDS of PP6C |
| PP6CCDS-R | GGTCGAGCCCGAGCCCTTGCTAGCGAGAAAGTAGGGCGCAATGGCCCG |  |
| PP6RCDS-F | TACACGAGAGACCGGATTGGTTGTATGCCTCTCTTAGACCAACGAAGC | To construct the CDS of PP6R |
| PP6RCDS-R | GGTCGAGCCCGAGCCCTTGCTAGCAGAAAGAGAAATCTCAAGTTCTCG |  |
| GDIHG-F | GCTGCAGCGGCCGCACAGTTTTTCGACTTGCTCAAGTTG | To construct the GDIHG‐specific plasmid |
| GDIHG-R | GCACACGATGCACGGCGCCGGCAC |  |
| GDYVDRG-F | GCTGCAGCGGCCGCAGCTGCCTACAACAGCGTGGAGACATTTGAG | To construct the GDYVDRG‐specific plasmid |
| GDYVDRG-R | CAGAAAAATGTAGTTTTGCTCCCC |  |
| GNHE-F | GCTGCAGCCGCGTCGCGGCAGATCACGACGGTGTAT | To construct the GNHE‐specific plasmid |
| GNHE-R | GCGCAGCAACGTAATGTGCCGGGG |  |
| HGG-F | GCGGCTGCACTGTCGCCGGACTTGAAGCTGCTC | To construct the HGG‐specific plasmid |
| HGG-R | CACGCAGAAGACTGAGTCGTCGAT |  |
| RG-F | GCGGCAGCCGGCTGGCTCTTTGGAGACAAAGTGGTGAAGCGCTTCAACCACCTGAA | To construct the RG‐specific plasmid |
| RG-R | CGGGTTCTCGGCCCATTCCTCGAC |  |
| H-F | GCACAACTCGCTATGGAGGGCTTTCGC | To construct the H‐specific plasmid |
| H-R | GGCGCGCGCGATCAGTTCGAGGCCA |  |
